# Polar cell membrane nanotubes containing microtubules and acidic vesicles render *Drosophila* eggs fertile

**DOI:** 10.1371/journal.pbio.3003533

**Published:** 2025-12-02

**Authors:** Sayan Acharjee, Banhisikha Saha, Neha Kumari, Jayeeta Nandi, Sudipta Adhya, Partha Protim Karmakar, Mohit Prasad

**Affiliations:** 1 Department of Biological Sciences, Indian Institute of Science Education & Research-Kolkata, Mohanpur Campus, Mohanpur, Nadia, West Bengal, India; 2 Laboratory of Malaria and Vector Research, National Institute of Allergy and Infectious Diseases, National Institutes of Health (NIH), Rockville, Maryland, United States of America; Whitehead Institute for Biomedical Research, UNITED STATES OF AMERICA

## Abstract

Membrane nanotubes serve as critical cytoskeletal structures that facilitate intercellular communication and signal transmission across distances in both plants and animals. Here, we report the role of microtubule (MT) nanotubes in rendering the *Drosophila* micropyle functional, a structure essential for sperm entry during fertilization. Our study highlights that MT-nanotubes emanate from the apical end of the specialized epithelial cells called the polar cells in late oogenesis, forming a narrow channel through the eggshell. Utilizing a combination of fly genetics, live cell imaging, and tissue immunochemistry, our research elucidates the structural and functional characteristics of the polar cell nanotube. We show that tubulin is vital for the formation of these nanotubes, which are enriched in the lateral membrane marker, Fasciclin III. Moreover, the overall polarity of the migrating border cell cluster is critical for the successful development of the micropyle. Notably, both lysosomal function and lysosomal trafficking within the polar cells are essential for the opening of the vitelline layer, further facilitating the micropyle’s role in fertilization.

## Abstract 2

Membrane nanotubes are cytoskeletal structures that facilitate intercellular communication. This study demonstrates that polar cell derived microtubule nanotubes play a role in *Drosophila* fertilization, creating a channel in the eggshell that facilitates sperm entry.

## Introduction

Membrane protrusions are specialized structures that play varied roles, including sensing the environment, generating traction force in migrating cells, and serving as signaling conduits between distant cells. In addition to aiding normal development and cellular homeostasis, cellular protrusions are also linked to the spread of various diseases [1]. Of all the diverse functions performed by membrane extensions, it is quite intriguing how intercellular communication between cells helps to generate complex forms in developing metazoans.

Membrane protrusions exhibit significant structural and functional diversity. They are referred to by different terms like Cytonemes, tunneling nanotubes (TNTs), membrane nanotubes, and microtubule-based nanotubes (MT-nanotubes) [2,3]. Though all the membrane protrusions mentioned above connect cells over long distances, Cytonemes are primarily actin-based while Nanotubes are mostly microtubule-based [4]. Functionally, Cytonemes form contacts with the target cell and primarily function in relaying signaling ligands/molecules [5]. In comparison, Nanotubes participate in a broader range of cellular activities, including organelle transport [6]. In general, long, narrow membrane protrusions (not exceeding 1.5 μm in width, extending beyond several cells) that are microtubule-based are commonly referred to as MT-Nanotubes [7,8]. If they are open and connect two cells, they can serve as a thoroughfare conduit for transporting vesicles, organelles, signaling molecules, and survival factors between them. Unfortunately, the open nanotubes aid the spread of prions, and there are instances where it is hijacked by pathogens to infect healthy cells [9]. Stressed tumor cells also utilize these structures for receiving survival factors from normal/healthy cells [10]. Intriguingly, TNTs in squamous carcinoma cells growing in a culture helps them to adapt to stress conditions [11–13].

On the other hand, closed nanotubes support paracrine or juxtracrine signaling [14]. In quite a few instances, they help sculpt an organ to render it functional. Like in the *Drosophila* male germline, the communication between the germline stem cells (GSC) and hub cells is aided by closed nanotubes that help retain stemness in the dividing GSC [15]. Similarly, long actin-based protrusions between fusing myoblasts assist in coordinating fly muscle development [16,17]. While, thin actin-based membrane extensions, referred to as Cytonemes, have been documented to transport morphogen signals to large distances and help coordinate *Drosophila* wing and eye development [4,18–20]. Thin membranous protrusions are also observed in zebrafish epiblast cells and developing Xenopus embryos that are believed to support material transfer between developing cells [21–23]. Though thin cellular protrusions are quite diverse, our understanding of their formation and function is unclear.

We have employed the *Drosophila* micropyle development as a model system to understand how the narrow thin membrane extension helps generate a pore in the eggshell to permit sperm entry during fertilization. The mature eggs in *Drosophila* have an external chorionic layer with two dorsal appendages at the anterior end. In between the two dorsal appendages is a small tubular extension of the chorionic layer called the micropyle. The micropyle comprises a vitelline part surrounded by a chorionic portion with a fine channel transversing through the chorionic portion. This open channel facilitates sperm entry during fertilization [24]. Beneath the micropylar opening lies the oocyte membrane, and once the sperm enters the micropyle, it directly fuses with the egg membrane [25]. Unlike termite eggs, *Drosophila* eggs have only one micropyle at the anterior end [26].

**Fig 1:**
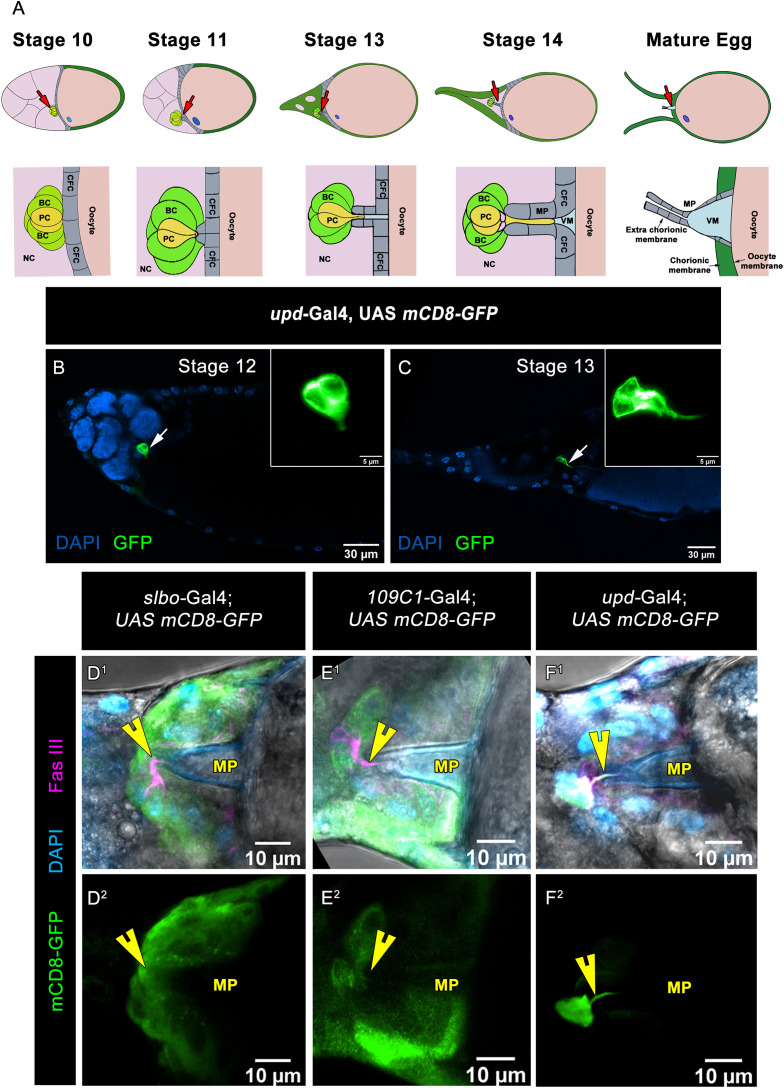
Characterisation of the polar cell protrusion formation at late oogenesis. **(A)** Schematic of stage wise tubular morphogenesis of the micropyle in late oogenesis. By stage 10, the border cell (BC) cluster has completed its migration and reaches the oocyte, while the centripetal follicle cells (CFCs) begin to migrate inward from both the dorsal and ventral sides of the egg chamber. These CFCs move to enclose the anterior region of the oocyte, creating a partition between the nurse cells (NCs) and the oocyte. At stage 11, CFC migration is completed, and they initiate the early steps of tubular morphogenesis of the micropyle. Concurrently, the polar cells (PCs), located centrally within the BC cluster, begin to form a protrusion directed toward the oocyte surface, marking the tip of the developing micropyle. From stage 13 onwards, this protrusion elongates into a narrow, thin extension that pierces the forming tubular structure, contributing to the creation of an open channel that serves as the conduit for fertilizing sperm. In a schematic representation, the nurse cells (NCs) are depicted in pastel purple, the oocyte in pastel magenta, border cells (BCs) in green, polar cells (PCs) in yellow, centripetal follicle cells (CFCs) in gray, and the vitelline membrane (VM) in cyan. **(B, C)** Different stages of egg chambers of indicated genotypes, DAPI (Blue), mCD8-GFP (Green), white arrows are marking polar cell and inset white boxes indicate the zoomed images of polar cell. **(D**^**1**^**–F**^**2**^) Stage 14 egg chambers of indicated genotypes, DAPI (Cyan), mCD8-GFP (Green), yellow arrow heads are marking polar cell protrusion. Please note that overexpression of mCD8-GFP with polar cell-specific *upd-*Gal4 only marks the protrusion which can suggest protrusion is the part of polar cell.

The micropyle has two structural components: the external tubular projection and the inner central channel. The morphogenesis of the micropyle starts at stage 10, when the centripetal follicle cells that surround the germline cells start to migrate along the junction of the nurse cell and oocyte. As they move, the morphogenesis of both the dorsal appendages and the micropyle is regulated by the Jun kinase (JNK) signaling [27]. Interestingly, we know that the open channel in the chorionic region of the micropyle is formed by a thin, narrow protrusion from the migrated border cell (BC) cluster [24]. The migratory BC cluster consists of 2 central polar cells (specialized follicle cells) surrounded by 6–8 migratory follicle cells. The BC cluster undergoes directed movement towards the oocyte in response to chemoattractants [28]. Concomitantly, the migrating centripetal cells initiate the tubular morphogenesis of the micropyle [27]. The end result of both events is the BC cluster occupying the tip of the micropyle protrusion. Stage 12 onwards, a narrow, thin protrusion from the polar cells pierces the developing micropyle, assisting the formation of an open channel (Fig 1A). This open channel is a prerequisite for fertilization as it serves as a conduit for fertilizing sperm.

Although the micropyle is crucial for determining the fertility of the egg chamber, surprisingly, this structure has not been explored in detail. There is no detailed information regarding the timing and the factors that assist in forming the polar cell protrusion. Since micropyle has wide implications in fertilization across an extensive range of species, understanding the molecular mechanism of this event is essential. It will also help us to understand how complex morphogenesis processes are coordinated to render an organ functional. In this study, we have characterized the polar cell protrusion formation event during micropyle development. We optimized the imaging conditions to visualize this polar cell shape transition in real-time and found it to be a highly dynamic structure supported primarily by microtubules. We found that polar cell protrusion formation is independent of successful migration and is directed towards the apical side of the BC cluster. We observed that lateral membrane proteins such as Fasciclin III (Fas III) and Coracle label the polar cell extension. We demonstrate that CEBP homolog, Slow border cells (Slbo), in the BCs non-cell autonomously plays a crucial role in the formation of polar cell protrusion. We found that the JNK signaling functions downstream of Slbo in the outer BCs to maintain their cluster polarity, which in turn facilitates the polar cell protrusion from the apical side. Coupling live cell imaging and genetics, we demonstrate that lysosomal function in the polar cell protrusion assists in clearing a passage through the vitellogenic membrane. Finally, we show that JNK signaling in the polar cells mediates the lysosomal trafficking toward the tip of the polar cell process, which is critical for sperm entry during fertilization.

## Results

### Characterization of the polar cell protrusion formation at late oogenesis

In the first step towards studying the polar cell protrusion, we labeled them by driving mCD8-GFP expression using *upd*-Gal4 (S1G–S1I Fig). The expression of mCD8-GFP is initiated in stage 2 egg chambers, labeling both anterior and posterior polar cells [29]. The expression of GFP is observed in the polar cells in late-stage egg chambers beyond 13. The polar cell protrusion was observed in early stage 13 egg chambers when the oocyte occupies approximately 70% of the whole egg chamber. The protrusion is visible until early stage 14 when the dorsal appendages are inside the nurse cell membrane, with occasionally one or two nurse cell nuclei persisting at the anterior end (Fig 1B and 1C). Since the two polar cells are centrally placed within 4–8 BCs (BCs), we were curious whether the BCs also physically contribute to the formation of polar cell protrusion. To verify this, we induced mCD8-GFP overexpression in the outer BCs using the BC specific driver *slbo-*Gal4 **(expression pattern in** S1A–S1C Fig)and *109C1*-Gal4 **(expression pattern in** S1D–S1F Fig). Unlike *upd*-Gal4, overexpression of mCD8-GFP in the outer BCs didn’t label the polar cell protrusion, suggesting that polar cells physically contribute to the polar cell process formation rather than the BCs (Fig 1D^1^–1F^2^).

Since the BC cells exhibit an asymmetric distribution of the polarity proteins, we mapped the location of the polar cell process with respect to the polarity of the migrating cluster. Once the BCs complete migration, the side facing the oocyte is enriched with apical, sub-apical polarity markers like Patj (Pals-associated tight junction) and Armadillo, respectively [30]. Strikingly, immunostaining of these apical proteins also lighted up two small circles (apical constrictions) above the polar cells in the center of the BC cluster, which radially distributes laterally between the BC-BC and BC-polar cell junctions. This distinct pattern of apical and subapical proteins is called the rosette pattern and is observed when the BC cluster is completely detached from the anterior follicle cell layer [30–32]. To check the identity of the polar cell process with respect to the polarity, we labeled the process by overexpressing GFP driven by *upd-*Gal4 and immunostaining with various polarity markers. Though the process was apically localized, we observed that neither Patj nor Armadillo protein was detected in the polar cell protrusion (S2A–S2F^2^ Fig). Strikingly, only the lateral markers, Coracle (Cora) and Fas III, labeled the polar process (S2J–S2O^2^ Fig). Cora is also enriched between the polar cell-polar cell (pc-pc) and BC-polar cell (bc-pc) junctions [30]. Fas III is present at the pc-pc junction right from the specification of polar cells and is observed as a single distinct band at the pc-pc junction in the migrating BC clusters [33]. In late oogenesis, Fas III is enriched at the apical edge of the polar cells, where the protrusion formation will initiate. Unlike Cora, Fas III is detected only between the two polar cells [33]. Since it also conspicuously marks the polar cell protrusion, it was used as a marker to label the protrusion in most of the experiments below. Altogether, we conclude that, unlike its apical origin, the polar cell process is enriched in lateral polarity proteins like Cora and Fas III.

**Fig 2:**
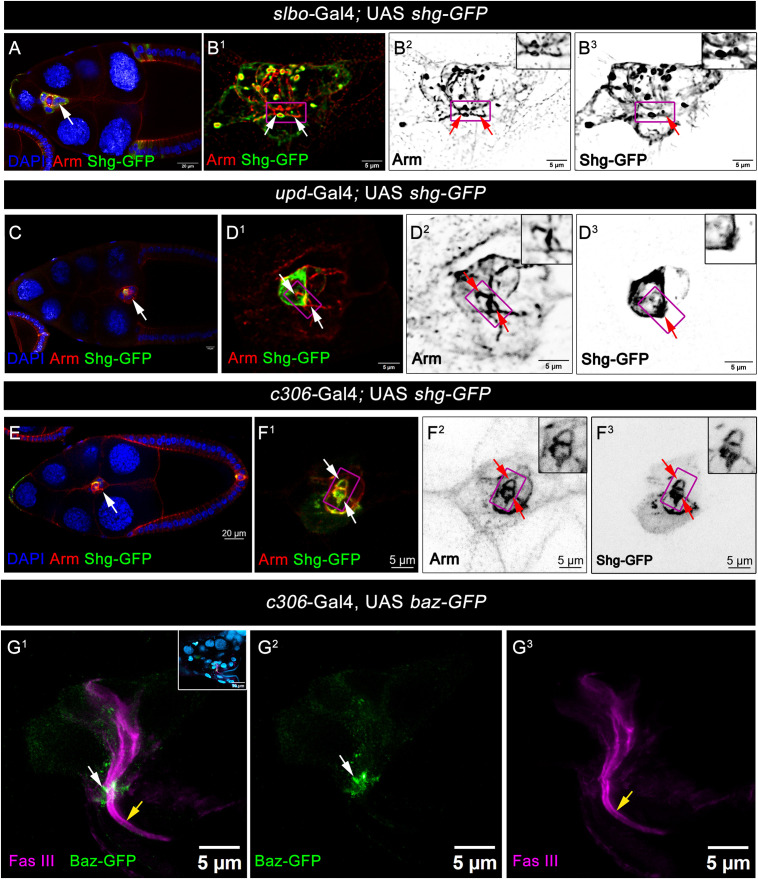
Border cell and Polar cell together contribute to form apical constriction of the migratory border cell cluster. **(A–B**^**3**^) Overexpression of the Shg-GFP with the border cell-specific *slbo*-Gal4 partially labeled the apical constriction. **(A)** Reference Stage 9 egg chambers of indicated genotype. Armadillo (Red), Shg-GFP (Green), DAPI (Blue). White arrow marks the border cell cluster. **(B**^**1**^**–B**^**3**^) Shg-GFP (Green) in the border cells colocalized with the Armadillo (Red). White arrows and magenta box mark the apical constriction of the border cell cluster. **(C–D**^**3**^) Overexpression of the Shg-GFP with the polar cell-specific *upd*-Gal4 partially labeled the apical constriction. **(C)** Reference Stage 9 egg chambers of indicated genotype. Armadillo (Red), Shg-GFP (Green), DAPI (Blue). White arrow marks the border cell cluster. **(D**^**1**^**–D**^**3**^) Shg-GFP (Green) in the polar cells colocalized with the Armadillo (Red). White arrows and magenta box mark the apical constriction of the border cell cluster. **(E–F**^**3**^) Overexpression of the Shg-GFP with the border cell cluster-specific *c306-*Gal4 (expressed in both polar cells and border cells) completely labeled the apical constriction. **(E)** Reference Stage 9 egg chambers of indicated genotype. Armadillo (Red), Shg-GFP (Green), DAPI (Blue). White arrow marks the border cell cluster. (**F**^**1**^**–F**^**3**^) Shg-GFP (Green) in the border cell cluster colocalized with the Armadillo (Red). White arrows and magenta box mark the apical constriction of the border cell cluster. **(G**^**1**^**–G**^**3**^) High-resolution 2D-STED image of polar cell protrusion at stage 13 egg chambers of indicated genotype. Where Fas III (Magenta) indicates polar cell protrusion and Baz-GFP (green) marks the apical constriction, white arrows mark the apical constriction of the cluster, and yellow arrows mark the polar cell protrusion.

Next, we were curious to examine the structure and dynamics of the polar cell process formation. Since the apical surface of the BC cluster has two conspicuous constrictions, we were curious if the polar cell process arose from them. To identify the precise location of the process, we labeled the polar cell process with Fas III in the background, where the apical protein, Bazooka-GFP (*Drosophila* Par3 homolog) is overexpressed in the migrating cluster. Using super-resolution STED imaging, we observed that the formation of polar cell protrusion was initiated between apical constrictions and extended towards the micropyle (Fig 2G^1^–2G^3^, S1 Movie). Altogether, our results above suggest that GFP-labeled polar cell protrusion is formed from the apical constriction, which sits like a cap over the micropyle.

We were interested in determining whether the observed apical constriction in the BC cluster is formed solely by the polar cells? The BC cluster consists of central polar cells surrounded by migratory BCs, so we sought to investigate this by overexpressing DE-Cadherin (Shotgun, Shg) in either the outer BCs or the polar cells independently to assess which cell type contributes to the apical constriction in the migrating cluster. We employed the Shg-GFP construct, where the Shg protein is physically tagged with GFP, and can be followed in real time for intracellular localization studies in organs and tissues [34]

Our hypothesis was that if the apical constriction were entirely formed by the polar cells, then Shg-GFP driven by *upd*-Gal4 (which is specifically expressed in the polar cells) would label the apical constriction. Conversely, if the apical constriction involved contributions from the BCs, Shg-GFP driven by *slbo*-Gal4 (which is expressed in the outer BCs) would label the constriction. We found that *upd*-Gal4-driven Shg-GFP partially labeled the apical constriction (Fig 2C–2D^3^), and similarly, *slbo*-Gal4-driven Shg-GFP also partially labeled the constriction (Fig 2A–2B^3^). These results suggest that both polar cells and BCs contribute to the formation of the apical constriction in the migratory BC cluster.

To further test this hypothesis, we overexpressed Shg-GFP using *c306*-Gal4 **(expression pattern in** S1J–S1L Fig), a driver that expresses in both the BCs and polar cells. Interestingly, *c306*-Gal4-driven Shg-GFP fully labeled the apical constriction (Fig 2E–2F^3^) supporting our earlier hypothesis that both polar cells and BCs together contribute to the formation of the polarized apical constriction in the migratory BC cluster.

### Polar cell protrusion is dynamic

**Fig 3:**
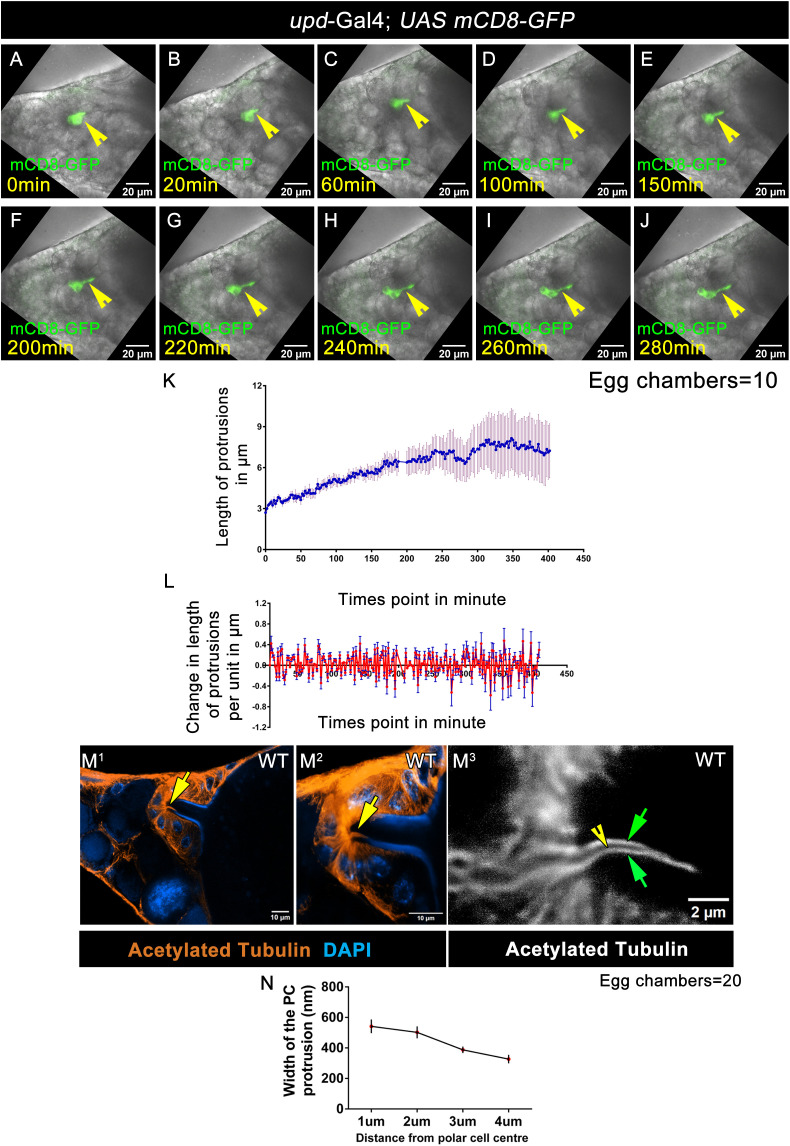
Polar cell protrusion is dynamic. **(A–J)** Time-lapse snapshot of stage 12 egg chambers of the indicated genotype. GFP (Green), Yellow arrowheads mark polar cell. **(K)** Quantitative analysis of the length of the polar cell protrusion at each time points. Error bars represent SEM. Detailed quantification in S1 Data. **(L)** Quantitative analysis of the delta changes of the length of the polar cell protrusion at each time points. Error bars represent SEM. Detailed quantification in S1 Data. **(M**^**1**^**–M**^**3**^) High-resolution 2D-STED image of polar cell protrusion at stage 13 WT egg chamber. **(M**^**1**^) Stage 13 reference image, DAPI (Blue), Acetylated-Tubulin (orange), and yellow arrow indicates the polar cell protrusion. **(M**^**2**^) Magnified 2D-STED images of polar cell protrusion with 40 nm resolution, Acetylated-Tubulin (orange), and yellow arrow indicates the polar cell protrusion. **(M**^**3**^) 2D-STED images of polar cell protrusion with 15 nm resolution, Acetylated-Tubulin (Gray), and green arrow indicate the detailed structure of polar cell protrusion. **(N)** Quantitative analysis of width of the polar cell protrusion from starting edge to the tip of the protrusion. Error bars represent SEM. Detailed quantification in S1 Data.

We found that the length of the polar cell protrusion is quite variable in similarly staged egg chambers. We conducted a real-time analysis to examine the reason for the above-observed variability. We standardized the fattening and imaging conditions to capture the polar cell protrusion in a large proportion (>60%) of egg chambers and observed it for 30 hours. We labeled the polar cell protrusion by overexpressing *mCD8-GFP* under the polar cell driver *upd-*Gal4. Post stage 10, the apical structure of the BC cluster is neo-laminated with the oocyte boundary. We performed live cell imaging in conjunction with differential interference contrast (DIC) microscopy to capture in real-time to how the polar cell protrusion is formed. In late stage 11 egg chambers, we observed two stable, thin protrusions extending from the polar cells towards the oocyte membrane. Around stage 12, the centripetal cells, in conjunction with the neo-laminated BC cluster, aid the formation of the micropylar structure, which develops as a projection of the oocyte membrane. Concomitantly, another thicker protrusion, probably due to the merger of thin protrusions from the two polar cells, extends towards the oocyte. We observed that the elongation of the polar cell protrusion was not continuous but exhibited intermittent extensions and retractions. By late stage 13, this thick protrusion assists in forming a narrow channel in the developing micropyle to facilitate sperm entry during fertilization (Fig 3A–3J, S2 Movie).

We measured the protrusion length frame by frame using ImageJ software. We observed the protrusion length extended up to a maximum of 7 µm **(2.70 µm ± 0.20 SEM at 0 min to 7.23 ± 1.98 SEM at 402 min,** Fig 3K, S1 Data). Though this length is mostly maintained with slight fluctuations, it was noticed that the growth of the protrusion was not linear. We plotted the difference in its length frame by frame and observed either positive or negative values at each time point (Fig 3L, S1 Data). This suggested that the protrusion formation is highly pulsatile and dynamic with alternative phases of extension and retraction. Next, we were curious to examine the molecular nature of the polar cell protrusion perse.

### Tubulin is indispensable for polar cell protrusion formation

We know that the active participation of cytoskeleton components is required to maintain the dynamic behavior of the polar cell protrusions. Though the existing literature suggests that both actin and tubulin are integral constituent of the polar cell protrusion (S3A^1^–S3B^2^ Fig), it is not clear which of these is indispensable for polar cell protrusion formation [35,36]. To determine their importance in protrusion formation, the assembly of actin and tubulin filaments was downregulated independently in the polar cells, and its effect on protrusion formation was examined.

Rac GTPases are one of the key regulators of actin dynamics that drive cellular membrane extensions. Downregulation of the Rac GTPase destabilizes the actin cytoskeleton and impedes membrane extension, including protrusions [37]. We downregulated Rac function by overexpressing a dominant negative form Rac1 in the polar cells, and quantified protrusion formation. We observed that polar cell protrusion formed and increased in length from Rac-depleted polar cells, similar to that observed in the control egg chambers **(*upd*-Gal4;UAS *GFP*;UAS *Rac1***^***N17***^**-8.60 µm ± 2.0 SEM, *n* = 5 egg chambers, *upd*-Gal4;UAS *GFP*/UAS *GFP*-8.52 µm ± 2.54 SEM, *n* = 5 egg chambers)** (S3C^1^–S3D^4^, S3F Fig, S8 Data, S2–S4 Movies). This was further validated by examining if the micropyle was open or closed in stage 14 egg chambers. It was observed that only 3% of the mature egg chambers had closed micropyle (*n* = 553 egg chambers) as compared to 1.47% observed in control egg chambers (*n* = 204). We recorded similar observations when a different isoform of Rac GTPase, Rac2, was depleted from the polar cells too **(*upd*-Gal4;UAS *GFP*;UAS *Rac2***^***RNAi***^**-8.65 µm ± 2.93 SEM, *n* = 4 egg chambers, *upd*-Gal4;UAS *GFP*/UAS *GFP*-7.53 µm ± 1.55 SEM, *n* = 5 egg chambers**, S3E^1^–S3E^4^, S3G Fig, S8 Data, S5 Movie). This suggests that Rac does not play a dominant role in forming polar cell protrusion, as interfering with its function does not block the micropylar channel in the mature eggs. To further investigate whether actin plays a significant role in the formation of polar cell protrusions, we downregulated the actin polymerizing proteins Diaphanous (Dia) and Enabled (Ena) specifically in polar cells. Strikingly, disruption of actin dynamics in the polar cells using the *upd*-Gal4 driver did not impair protrusion formation. In fact, **94.93% ± 2.31 SEM** and **93.53% ± 0.71 SEM** of stage 14 egg chambers exhibited polar cell protrusions upon Dia and Ena depletion, respectively (*n* = 350 and 233 egg chambers), which is comparable to the **95.72% ± 0.89 SEM** observed in control egg chambers (*n* = 305). These results suggest that actin polymerization is largely dispensable for polar cell protrusion formation (S4A^1^–S4D Fig, S9 Data).

**Fig 4:**
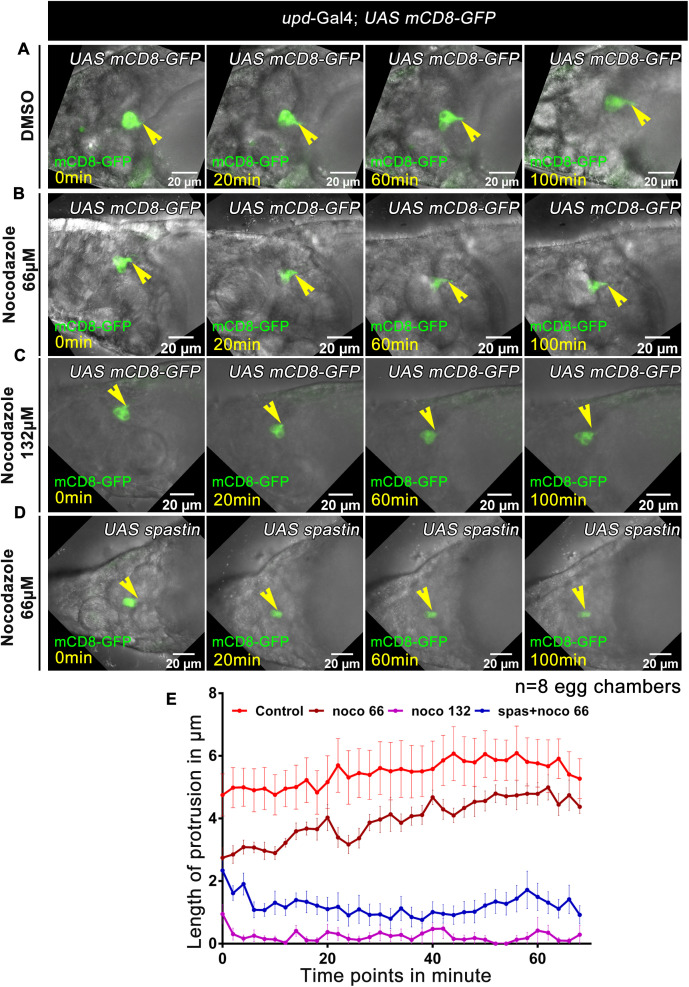
Tubulin is indispensable for polar cell protrusion formation. **(A–D)** Time-lapse snapshot of stage 12 egg chambers of the indicated genotypes. **(A)** Time-lapse snapshot of DMSO-treated stage 12 egg chambers of the indicated genotype. **(B**) Time-lapse snapshot of 66 µM Nocodazole-treated stage 12 egg chambers of the indicated genotype. **(C)** Time-lapse snapshot of 132 µM Nocodazole-treated stage 12 egg chambers of the indicated genotype. **(D)** Time-lapse snapshot of 66 µM Nocodazole-treated stage 12 egg chambers of the indicated genotypes. GFP (Green), Yellow arrowheads mark polar cell. **(E)** Quantitative analysis of the length of the polar cell protrusion at each time points. Error bars represent SEM. Detailed quantification in S2 Data.

Microtubules consist of heterodimer repeats of alpha and beta tubulin monomers, and to investigate if they are required in the formation of polar cell protrusion, we attempted to disrupt their assembly in the polar cells [38]. Nocodazole is a microtubule depolymerizing drug that has been widely used as an antineoplastic agent [39,40]. Nocodazole promotes hydrolysis of GTP bound to the free alpha-beta tubulin heterodimer and reduces its affinity towards the existing microtubule chain [39]. The high concentration of this drug in µM range induces rapid destabilization of the microtubule network in cell culture systems [41]. As we worked out the effect of different concentrations of Nocodazole, we observed the polar cell protrusion formation was severely inhibited in the presence of 132 µM of Nocodazole. In real-time, we observed that egg chambers treated with inhibitor extended short polar cell protrusions, which immediately retracted **(Nocodazole 132 µM treated *upd*-Gal4;UAS *mCD8*-*GFP*;UAS *mCD8*-*GFP*-0.28 µm ± 0.28 SEM, *n* = 8 egg chambers)** (Fig 4C, S8 Movie). Importantly, the replacement of the Nocodazole-supplemented medium with the normal medium rescued the stalled polar cell protrusion formation, suggesting that the effect of Nocodazole was reversed and the effect observed by the inhibitor was not an outcome of the toxicity to the developing egg. To check the specificity of the Nocodazole on the polar cell protrusion perse, we coupled genetics with inhibitor treatment and captured the polar cell protrusion formation under live conditions. *Drosophila* encodes several molecules that depolymerize microtubules: Stathmin, Katanin, Fidgetin, Klp10A, and Spastin. Spastin initiates microtubule depolymerization at the minus end and plays an important role in mitosis and axonal transport [42]. We overexpressed Spastin in the polar cells in the background, where egg chambers were incubated in a lower concentration of Nocodazole. Strikingly, overexpression of Spastin coupled with 66 µM of Nocodazole treatment completely blocked the polar cell protrusion **(Nocodazole 66 µM treated *upd*-Gal4;UAS *mCD8*-*GFP*;UAS *Spastin*-0.92 µm ± 0.28 SEM, *n* = 8 egg chambers)** (Fig 4D, S9 Movie) as observed above for 132µM of Nocodazole treatment (Fig 4C, S8 Movie). However, overexpression of Spastin alone **(*upd*-Gal4;UAS *mCD8*-*GFP*;UAS *spastin*-6.56 µm ± 0.63SEM, *n* = 5 egg chambers, *upd*-Gal4;UAS *mCD8*-*GFP*;UAS *mCD8-GFP*-5.27 µm ± 0.63SEM, *n* = 5 egg chambers)**, nor independent treatment of egg chambers with 66 µM of Nocodazole, didn’t impede the polar cell protrusions**(Nocodazole 66 µM treated *upd*-Gal4;UAS *mCD8*-*GFP*;UAS *mCD8*-*GFP*-4.37 µm ± 0.21 SEM, *n* = 8 egg chambers)** (S5B^1^–S5C, Figs 4B; S7, S10, S11 Movies). It was pleasing to observe that the synergistic effect of a lower concentration of Nocodazole and Spastin overexpression completely inhibited the formation of polar cell protrusions. The dynamics of polar cell protrusion of all conditions are represented graphically in Fig 4E, S2 Data, S5C Fig, S10 Data. Altogether, our results above demonstrate that microtubules play a significant role during polar cell protrusion formation.

Given that the microtubules are rigid structures, we were curious about how the polar cell process exhibited dynamic behavior. We know that acetylation of the α subunit of microtubule renders it flexible and stable. Thus, we immunostained the egg chambers with acetylated tubulin antibody and found that acetylated tubulin marked the edges of the polar cell process with a lumen within. The width of the lumen gradually decreased from **595.5 nm ± 23.38 SEM** observed at the base of the process to **333 nm ± 10.43 SEM** measured at the tip (Fig 3M^1^–3N, S1 Data). Based on this, we conclude that the polar cell process is a nanotube structure.

Having determined that the microtubule is important for polar cell protrusion formation and that its orientation is directed towards the oocyte, we were curious to identify factors responsible for process formation.

### Slbo in BC non-cell-autonomously regulates polar cell protrusion formation

**Fig 5:**
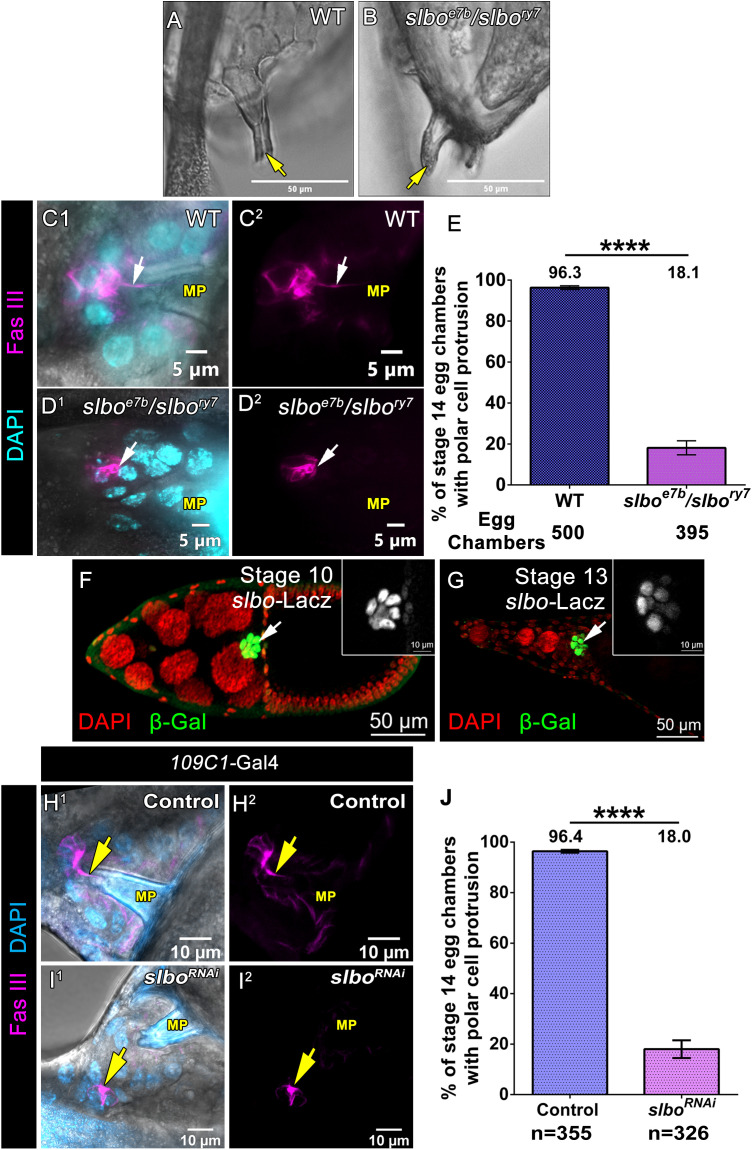
Slbo in border cell non-cell-autonomously regulates polar cell protrusion formation. **(A, B)** DIC images of stage 14 egg chambers of indicated genotypes. Yellow arrows mark the micropyle cone. **(C**^**1**^**–E)** Heteroallelic *slbo* mutant affects polar cell protrusion formation. **(C**^**1**^**–D**^**2**^) Stage 13 egg chamber of indicated genotypes, Fas III (Magenta) and DAPI (Cyan). White arrows indicate polar cell protrusion. **(E)** Quantification of % of stage 14 egg chambers with polar cell protrusion. Error bars represent SEM, nonparametric *t* test, ****P < 0.0001. Detailed quantification in S3 Data. **(F, G)** *slbo- Lacz* expression pattern in *Drosophila* egg chambers. **(F)** Stage 10 egg chambers where *slbo- Lacz* is highly expressed in the border cell cluster. **(G)** Stage 13 egg chambers where *slbo- Lacz* is highly expressed in the border cell cluster and centripetal cells. *slbo- Lacz* (Green), DAPI (Red) white arrows mark *slbo- Lacz* expression pattern, inset white box indicates Slbo positive cells. **(H**^**1**^**–J)** Downregulation of Slbo function in the outer border cells affects polar cell protrusion formation. **(H**^**1**^**–I**^**2**^) Stage 13 egg chamber of indicated genotypes, Fas III (Magenta) and DAPI (Cyan). Yellow arrows indicate polar cell protrusion. **(J)** Quantification of % of stage 14 egg chambers with polar cell protrusion. Error bars represent SEM, nonparametric *t* test, ****P < 0.0001. Detailed quantification in S3 Data.

We were drawn to the heteroallelic combination of two *slbo* mutants: *slbo*^*ry7*^ and *slbo*^*e7b*^ that is known to result in the closed micropyle in mature eggs (Fig 5A and 5B). When we stained the polar cell protrusions in the *slbo*^*ry7*^/*slbo*^*e7b*^ egg chambers, we found them to be completely lacking the protrusion (**WT-96.37% ± 0.86 SEM, *n* = 500 egg chambers and *slbo***^***ry7***^***/slbo***^***e7b***^***-*18.14% ± 3.42 SEM**, ***n* = 395 egg chambers,** Fig 5C^1^–5E, S3 Data). Since the *slbo*^*ry7*^/*slbo*^*e7b*^ egg chambers exhibit BC migration defect, it intrigued us to check if the physical contact between BC cluster and the micropyle was a requisite for polar cell protrusion formation. We know that at stage 10B of *Drosophila* oogenesis, proper neo-lamination of the BC cluster with the oocyte boundary is necessary to maintain physical contact between the BC cluster and micropyle. Previous studies have shown that disrupting gap junction function within the BC cluster and nurse cells impairs neo-lamination [43]. Thus, we downregulated Innexin 4 (Zpg) function in the germline by overexpressing *zpg RNAi* using the *mat-*α *tubulin*-Gal4 driver and examined the polar cell protrusion formation. Though downregulation of Zpg in the nurse cells disrupted the physical contact between the BC cluster and the micropyle it did not impede the formation of polar cell protrusion. We observed long polar cell protrusions formed and interestingly it was directed towards the micropyle, despite the cluster being far from the micropyle (S6A^1^–S6B^2^ Fig). This result suggests that the physical contact between the BC cluster and the micropyle is not essential for forming polar cell protrusions.

Next, we were curious to decipher which cells of the developing egg chamber required the Slbo function to form polar cell protrusion. Slbo is known to be expressed in both the cells of the migrating cluster (border and polar cells) and the centripetal follicle cells (Fig 5F and 5G). To delineate the function of the Slbo in different cell populations with respect to polar cell protrusion formation, we downregulated its function using the RNAi approach and examined the status of process formation. We observed Slbo depletion in the polar cells (*upd*-Gal4) or the centripetal cells (*c289b11*-Gal4, **expression pattern in** S1M–S1N Fig, [44]) didn’t impede the process formation, as 94.4% of egg chambers with Slbo depleted polar cells exhibited protrusion (*n* = 280 egg chambers) and 93% of egg chambers with Slbo depleted centripetal cells exhibited protrusion (*n* = 210 egg chambers) similar to that 95.9% and 94.1% observed in the control respectively (*n* = 250 egg chambers, 270 egg chambers respectively) **(*upd*-Gal4; UAS *dicer II*-95.91% ± 1.19 SEM, *n* = 250 egg chambers and *upd*-Gal4; UAS *slbo RNAi-*94.4% ± 1.89 SEM**, ***n* = 280 egg chambers and *c289b11*-Gal4; UAS *dicer II*-94.14% ± 0.72 SEM, *n* = 270 egg chambers and *c289b11*-Gal4; UAS *slbo RNAi-*93% ± 1.01 SEM**, ***n* = 210 egg chambers** S7D^1^–S7I Fig, S11 Data). Strikingly downregulation of *slbo* function in the BCs (*slbo*-Gal4) impaired polar cell protrusion formation, with only 24.1% of egg chambers exhibiting the polar cell protrusion formation (*n* = 380 egg chambers) compared to the 95.2% observed in the controls (*n* = 450 egg chambers) **(*slbo*-Gal4; UAS *dicer II*-95.25% ± 1.61 SEM, *n* = 450 egg chambers and *slbo*-Gal4; UAS *slbo RNAi-*24.1% ± 3.86 SEM**, ***n* = 380 egg chambers** S7A^1^–S7C Fig, S11 Data). Since Slbo Gal4 is also expressed in the centripetal cells, we used another outer BC specific Gal 4 (*109C1-*Gal4, S1D–S1F Fig) to down-regulate *slbo* function in the outer BCs to check its effect on polar cell protrusion. Satisfyingly, we observed that knockdown *slbo* in the outer BCs impaired polar cell formation, as 18.0% of egg chambers (*n* = 326 egg chambers) exhibited PC protrusion formation compare to the control 96.4% (*n* = 355 egg chambers) **(*109c1*-Gal4; UAS *dicer II*-96.4% ± 0.55 SEM, *n* = 355 egg chambers and *upd*-Gal4; UAS *slbo RNAi-*18.0% ± 3.52 SEM**, ***n* = 326 egg chambers** Fig 5H^1^–5J, S3 Data). Overall, our data above suggested that Slbo function in the outer BC non-cell autonomously promotes polar cell protrusion formation from the central polar cells (S7J Fig). This observation intrigued and challenged us to examine how Slbo function in the outer BCs stimulated the adjacent polar cell to extend the micropylar process.

### The polarity of BC cluster required for polar cell protrusion formation

**Fig 6:**
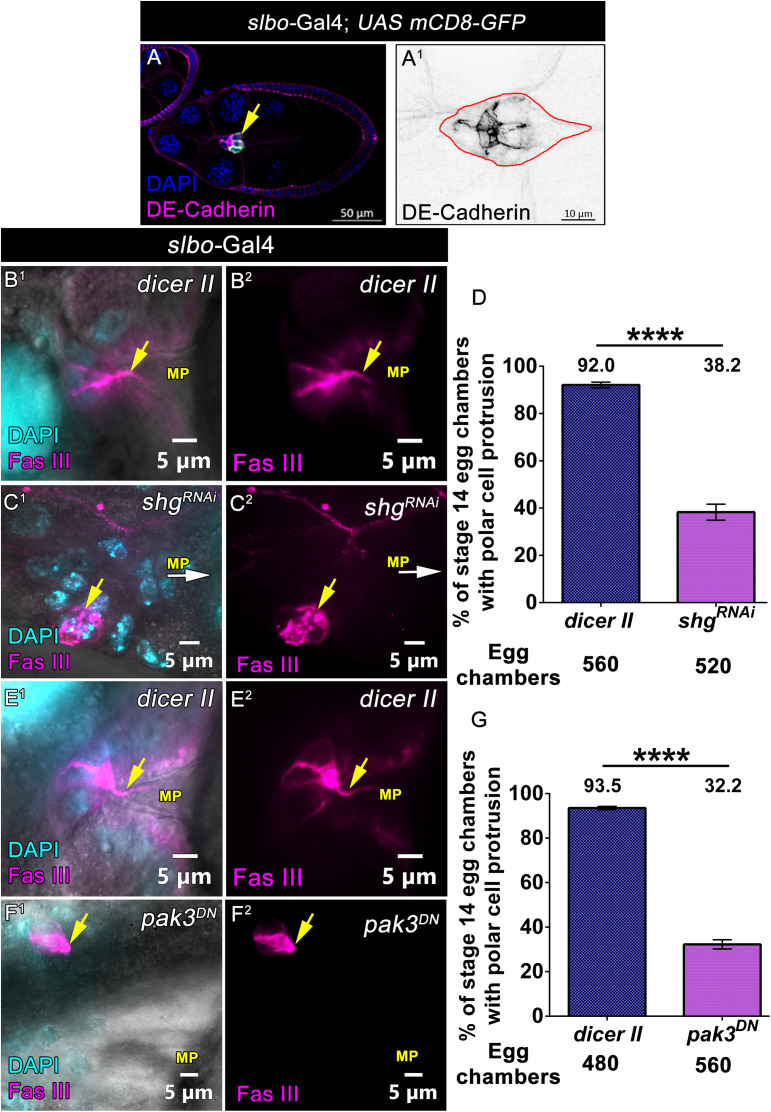
The polarity of border cell cluster required for polar cell protrusion formation. **(A–A**^**1**^) Distribution of DE-Cadherin in the migratory border cell cluster. **(A)** Stage 9 egg chambers of indicated genotype. DE-Cadherin (Magenta), DAPI (Blue), yellow arrow marks the migratory border cell cluster. **(A**^**1**^) DE-Cadherin distribution in the magnified border cell cluster. Where DE-Cadherin localized in between PC-PC, PC-BC, and BC-BC. DE-Cadherin (Black), Red line indicates the outline of BC cluster. **(B**^**1**^**–D)** Downregulation of Shotgun function in the border cells affects polar cell protrusion formation. **(B**^**1**^**–C**^**2**^) Stage 13 egg chamber of indicated genotypes, Fas III (Magenta) and DAPI (Cyan). Yellow arrows indicate polar cell protrusion, white arrows indicate the direction of MP. **(D)** Quantification of % of stage 14 egg chambers with polar cell protrusion. Error bars represent SEM, nonparametric *t* test, ****P < 0.0001. Detailed quantification in S4 Data. **(E**^**1**^**–G)** Downregulation of Pak3 function in the border cells affects polar cell protrusion formation. **(E**^**1**^**–F**^**2**^) Stage 13 egg chamber of indicated genotypes, Fas III (Magenta) and DAPI (Cyan). Yellow arrows indicate polar cell protrusion. **(G)** Quantification of % of stage 14 egg chambers with polar cell protrusion. Error bars represent SEM, nonparametric t *t*est, ****P < 0.0001. Detailed quantification in S4 Data.

Our earlier results suggest that outer BCs communicate with the inner polar cells to facilitate the formation of polar cell protrusion. Since the border and polar cells adhere to each other, we examined whether disrupting the cell adhesion between them is required for polar cell protrusion formation. Since *Drosophila* E-Cadherin, a subapical cell adhesion protein, is known to mediate BC and polar cell adhesion [45] (Fig 6A–6A^1^), we downregulated E-Cadherin function in the outer BCs by RNA interference approach. Though the cluster was still intact, we observed that BC overexpressing E-Cadherin RNAi exhibited very low levels of E-Cadherin protein (S8A^1^–S8B^2^ Fig). Interestingly, we observed that the depletion of E-Cadherin in the outer BC cluster by the driver *slbo*-Gal4 impaired polar cell protrusion formation, as 38.2% of stage 14 egg chambers exhibited polar cell protrusion (*n* = 520 egg chambers) compared to 92% observed in the control (*n* = 560 egg chambers) **(*slbo*-Gal4; UAS *dicer II*-92.08% ± 1.20 SEM, *n* = 560 egg chambers and *slbo*-Gal4; UAS *shg RNAi −*38.26% ± 3.36 SEM**, ***n* = 520 egg chambers,** Fig 6B^1^–6D, S4 Data). This result helped us conclude that the function of E-Cadherin between the outer BCs and the inner polar cells is essential for protrusion formation. Overall, our results suggest that E-Cadherin-mediated adhesion between the border and polar cells is critical for the overall polarity of the migrating cluster. Thus, we were curious if the cluster polarity was a prerequisite for polar cell protrusion formation. We examined the polar cell protrusions in BC clusters with disrupted polarity to test this possibility. Previously, we have shown that p21 Activated Kinase 3 (Pak3), a serine/threonine protein kinase, regulates the asymmetric distribution of the polarity proteins in the migratory BC cluster [30]. As Pak3-depleted clusters exhibit disrupted polarity, we examined the polar cell protrusion formation in the background where dominant negative Pak3 was overexpressed in the BCs. As per our expectation, we observed only 32.2% of Pak3 depleted BCs exhibited protrusion formation (*n* = 560 egg chambers) compared to 93.5% observed in the control (*n* = 480 egg chambers) **(*slbo*-Gal4; UAS *dicer II*-93.53% ± 0.71 SEM, *n* = 480 egg chambers and *slbo*-Gal4; UAS *pak3 DN-*32.26% ± 2.07 SEM**, ***n* = 560 egg chambers,** Fig 6E^1^–6G, S4 Data). We also observed that downregulation of Pak3 affects the distribution of Armadillo in the migratory BC cluster (S8C and S8D Fig). Our data suggest that the distinct distribution of polarity proteins facilitates the formation of polar cell protrusions. Next, we were curious to examine the molecular basis of polarity required for polar cell protrusion formation.

### Slbo functions through JNK to modulate the formation of polar cell protrusion

**Fig 7:**
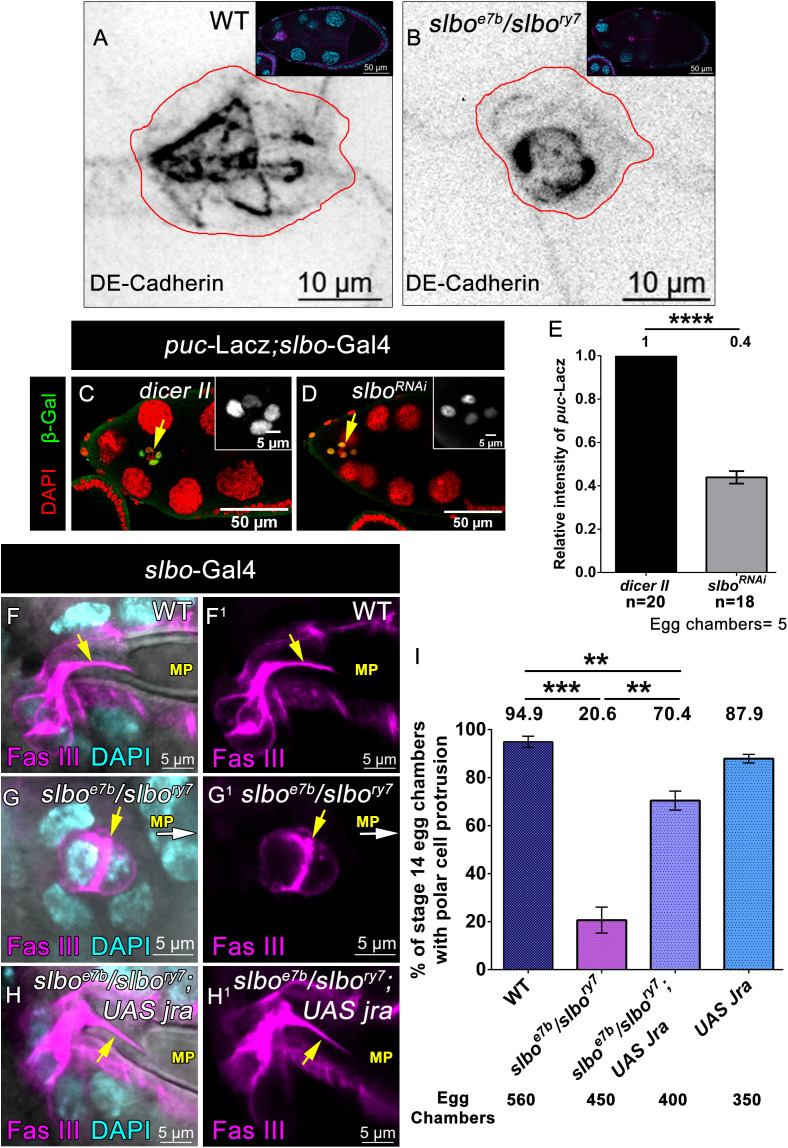
JNK signaling, Downstream of Slbo facilitates polar cell protrusion formation. **(A, B)** Heteroallelic *slbo* mutant affects the distribution of DE-Cadherin in the migratory border cell cluster. Where DE-Cadherin localized in between PC-PC, PC-BC, and BC-BC in the control which was mis-localized in the Slbo-depleted cluster. DE-Cadherin (Black), red lines mark the outline of BC cluster and inset box represent the reference egg chambers. **(C–E)** Downregulation of Slbo expression in the border cells affect *puc- Lacz* expression. **(C–D)** Stage 10 egg chambers where *puc- Lacz* is expressed in the border cell cluster. *puc- Lacz* (Green, inset gray), DAPI (Red) yellow arrows mark *puc- Lacz* expression pattern, inset white box indicates *puc- Lacz* positive cells. **(E)** Quantification of the relative intensity of *puc- Lacz* in the border cells. Error bars represent SEM, nonparametric *t* test, ****P < 0.0001, *n* = Total no. of border cells. Detailed quantification in S5 Data. **(F–I)** Overexpression of Jra in the *slbo* mutant border cells rescues the *slbo* mutant phenotype. **(F–H**^**1**^) Stage 13 egg chamber of indicated genotypes, Fas III (Magenta), and DAPI (Cyan). Yellow arrows indicate polar cell protrusion and white arrows indicate the direction of MP. **(I)** Quantification of % of stage 14 egg chambers with polar cell protrusion. Error bars represent SEM, nonparametric *t* test, ***P < 0.001, **P < 0.01. Detailed quantification in S5 Data.

Slbo is the transcription factor required for the specification and migration of the BC towards the oocyte. Given that Slbo depleted BCs impede polar cell protrusion formation, we investigated how Slbo in BCs non-cell autonomously regulates this process. We examined the polarity of Slbo-depleted migrating BCs by examining DE-Cadherin distribution in heteroallelic *slbo*^*e7b*^*/slbo*^*ry7*^ combination of egg chambers. Since Slbo mutants also affect the specification of BCs, we focused on clusters with more than five BCs. In these clusters, we observed a significant alteration in DE-Cadherin distribution in Slbo mutant BCs compared to the control (Fig 7A and 7B). To further confirm the role of Slbo in BCs, we downregulated Slbo expression by overexpressing Slbo RNAi using *slbo*-Gal4. Upon examining the distribution of DE-Cadherin, we observed a clear alteration in the distribution of DE-Cadherin in the *slbo* knockdown BCs too (S9A and S9B Fig), suggesting that Slbo is essential for proper adhesion and maintenance of polarity of the whole cluster. Given that JNK signaling mediates localization of the apical marker Bazooka and previous studies demonstrating the JNK pathway is critical for dorsal appendage and micropyle formation, we were prompted to investigate the status of JNK signaling in Slbo-depleted clusters [46], Puckered is a downstream target of the JNK signaling and is routinely used to access its status in various developmental contexts [47]. We employed *puc*-LacZ construct and observed a distinct reduction in its expression in the BCs **(*puc*-Lacz; *slbo*-Gal4/*slbo***^***RNAi***^**-0.4 ± 0.02 SEM)** compared to that observed in control **(*puc*-Lacz/UAS *dicer II*; *slbo*-Gal4-1 ± 0.02 SEM)** (Fig 7C–7E, S5 Data). This suggests that JNK signaling is impaired in the Slbo-depleted BC clusters.

Next, we were curious if JNK signaling per se in the BCs regulates polar cell protrusion formation. We disrupted the JNK pathway by overexpressing Basket DN and Kayak RNAi using *slbo*-Gal4. Both Basket and Kayak are integral components of JNK pathway. Basket (Bsk) is a serine-threonine kinase that phosphorylates and activates transcription factor AP-1, a heterodimer of Jun-related antigen (Jra, also known as c-Jun) and Kayak (Kay, also known as Fos), to stimulate the expression of JNK genes [46,47]. We observed that the downregulation of both Bsk and Kayak independently in the BCs led to impaired polar cell protrusion formation. We observed only 40% of Kayak depleted BCs exhibiting the polar cell protrusion (*n* = 280 egg chambers) and 28.6% of Bsk depleted BCs exhibiting the polar cell protrusion (*n* = 430 egg chambers) compared to the 94.5% observed in the controls (*n* = 355 egg chambers) **(*slbo*-Gal4; UAS *dicer II*-94.54% ± 0.76 SEM, *n* = 355 egg chambers and *slbo*-Gal4; UAS *Kayak RNAi-*40.06% ± 3.35 SEM**, ***n* = 280 egg chambers, *slbo*-Gal4; UAS *bsk DN-*28.68% ± 4.23 SEM**, ***n* = 430 egg chambers,** S9F^1^–S9I Fig, S12 Data). This suggests that JNK signaling in the outer BC is crucial for the polar cell protrusion formation.

Finally, we were curious to check if the impaired JNK signaling was indeed the cause for the absence of polar cell protrusion observed in the *slbo* mutant egg chambers. We know that JNK activation stimulates the transcription factor Jun-related Antigen (D-Jun) to regulate the expression of JNK target genes, which are essential for regulating cellular processes such as cell migration and polarity [46,48,49]. To test our hypothesis, we overexpressed the transcription factor Jra in Slbo-depleted BCs. Remarkably, we observed that overexpression of Jra in the *slbo* mutant BCs rescued polar cell protrusion formation. Strikingly, we observed 70.4% of the *slbo* mutant egg chambers over expressing Jra in the BCs (*slbo*^*e7b*^ *slbo*-Gal4/*slbo*^*ry7*^; UAS *Jra*, *n* = 400 egg chambers) exhibiting the polar cell protrusion compared to 20.6% observed in the *slbo*^e7b^/*slbo*^*ry7*^ (*n* = 450 egg chambers). In control, we observed 94.9% egg chambers exhibiting polar cell protrusion (*n* = 560 egg chambers) **(WT-94.93% ± 2.31 SEM, *n* = 560 egg chambers and *slbo***^***ry7***^***/slbo***^***e7b***^***-*20.61% ± 5.40 SEM**, ***n* = 450 egg chambers, *slbo***^***ry7***^***/slbo***^***e7b***^ ***slbo*-Gal4; UAS *jra-*70.44% ± 3.97SEM**, ***n* = 400 egg chambers, *slbo*-Gal4; UAS *jra-*87.9% ± 1.80SEM**, ***n* = 350 egg chambers,** Fig 7F–7I, S5 Data). Also, we checked the polarity of migrating cluster in this rescue background, and interestingly, we observed that overexpression of Jra in *slbo* mutant outer BCs rescues the distribution of Armadillo in the migrating cluster (S9C–S9E Fig). Overall, our data suggest that JNK signaling, which is downstream of Slbo in the outer BCs, modulates the cluster polarity, which in turn is critical for the formation of polar cell protrusion.

Next, we were curious to understand how polar cell protrusion facilitates the formation of functional micropyle.

### Lysosomal component of the Polar cell facilitates the Micropyle channel opening

**Fig 8:**
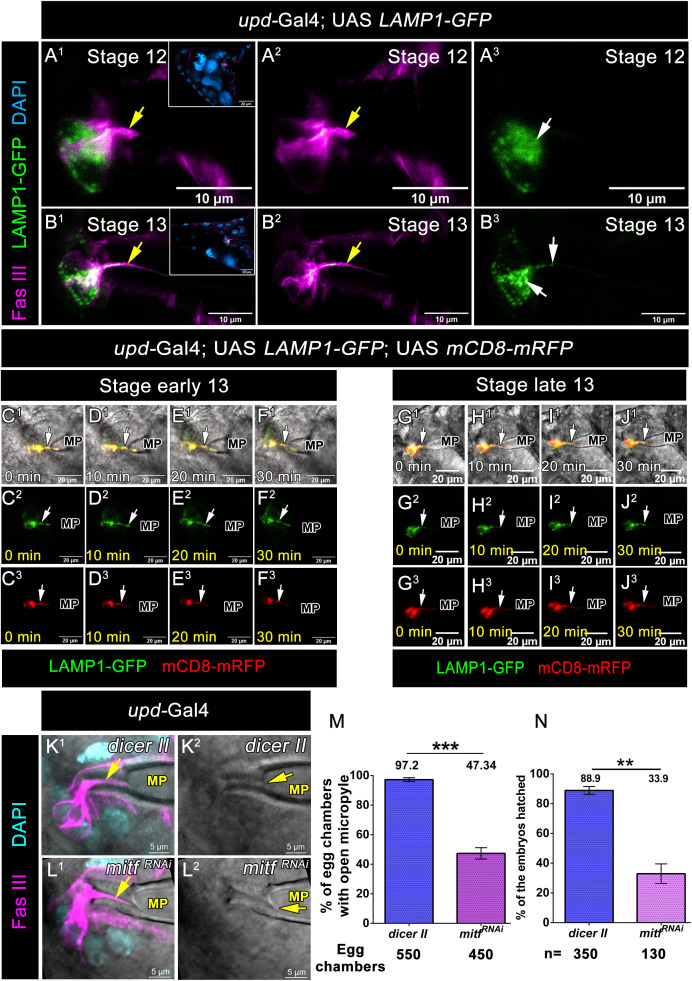
Lysosomal component of the Polar cell facilitates the Micropyle channel opening. **(A**^**1**^**–B**^**3**^) LAMP1-GFP expression pattern in the polar cells of indicated genotypes. **(A**^**1**^**–A**^**3**^) Stage 12 egg chambers of indicated genotypes. Fas III (Magenta), LAMP1-GFP (Green), yellow arrows are marking polar cell protrusion, and white arrow marks the LAMP1-GFP puncta which is localized mostly in the apical side and inset white boxes indicate the reference image of the stage 12 egg chamber. **(B**^**1**^**–B**^**3**^) Stage 13 egg chambers of indicated genotypes. Fas III (Magenta), LAMP1-GFP (Green), yellow arrows are marking polar cell protrusion, and white arrow marks the LAMP1-GFP puncta which is localized mostly in the apical side, also present in the polar cell protrusion and inset white boxes indicate the reference image of the stage 13 egg chamber. **(C**^**1**^**–F**^**3**^) Time-lapse snapshot of lysosomal movements through the nanotube at early stage 13 egg chamber of the indicated genotype. LAMP1-GFP (Green), mCD8-mRFP (Red), white arrows mark polar cell protrusion. **(G**^**1**^**–J**^**3**^) Time-lapse snapshot of lysosomal movements through the nanotube at late stage 13 egg chamber of the indicated genotype. LAMP1-GFP (Green), mCD8-mRFP (Red), white arrows mark polar cell protrusion. **(K**^**1**^**–M)** Downregulation of Mitf function in the polar cells affects micropyle channel opening. **(K**^**1**^**–L**^**2**^) Stage 13 egg chamber of indicated genotypes, Fas III (Magenta), and DAPI (Cyan). Yellow arrows indicate polar cell protrusion **(K**^**1**^**, L**^**1**^) and open micropyle channel **(K**^**2**^**, L**^**2**^). **(M)** Quantification of % of chambers with open micropyle. Error bars represent SEM, nonparametric *t* test, ***P < 0.001. Detailed quantification in S6 Data. **(N)** Quantification of % of the embryos hatched after 30 hours of egg laying. Error bars represent SEM, nonparametric *t* test, **P < 0.01. Detailed quantification in S6 Data.

Lukas H. Margaritis (1984) demonstrated that the entire micropyle cone consists of an inner vitelline protrusion and an outer hollow endo-chorionic cylinder containing the canal [24]. Our live-cell imaging data demonstrated that the polar cell protrusion gradually elongated towards the oocyte, and in late-stage 13 egg chambers, it connected with the inner vitelline membrane. This observation prompted us to investigate the functionality of the polar cell protrusion and its role in the formation of the micropyle channel. Based on its diameter, polar cell protrusion is a nanotube-like structure resembling a TNT or Cytoneme. Unlike, TNTs that are typically open at both ends, the polar cell protrusion is closed. This raises two possibilities: the protrusion might secrete molecules that aid in the digestion of the vitelline membrane, or it could fuse with the vitelline membrane, facilitating the formation of the lumen within the micropyle channel. The transmembrane pH is a key factor in lumen and pore formation [50–52]. To investigate this, we incubated the egg chambers with Lysotracker. Since the micropyle cone was labeled with Lysotracker, it suggested that it is acidic in nature (S10A^1^–S10A^2^ Fig). This finding prompted us to explore the relationship between the polar cell protrusion and the acidic pH of the micropyle. Lysosomes are key regulators of the acidic environment within cells. To further investigate the role of lysosomes in the polar cell protrusion, we examined the status of Lysosome-associated membrane protein 1 (LAMP1) in fixed samples. LAMP1 is a lysosomal marker that can provide insights into lysosomal distribution and function [53]. We overexpressed *LAMP1-GFP* using a polar cell-specific *upd*-Gal4 driver and, in this background, stained for Fas III, which marks the polar cell protrusions. In stage 12, when the polar cells begin to initiate protrusion formation, we observed that LAMP1-GFP was diffuse, with enrichment in the apical and lateral side of the polar cells. We also observed LAMP1 was localized at the polar cell junctions, with some GFP puncta present at the basal side (Fig 8A^1^–8A^3^). By stage 13, when the protrusions had fully elongated, LAMP1 puncta were localized within the polar cell protrusions (Fig 8B^1^–8B^3^). This dynamic distribution of LAMP1 suggested a potential role for lysosomal activity in the formation and function of the polar cell protrusions, particularly in relation to the acidic environment within the micropyle cone.

The differential localization of LAMP1 was intriguing, prompting us to investigate its dynamic trafficking further. We performed live-cell imaging to track LAMP1 movement through the polar cell protrusion. We captured early stage 13 egg chambers, where LAMP1 was predominantly localized to the apical side in the background where the polar cell protrusion were labeled using mCD8-RFP. Initially, we observed that all LAMP1-positive puncta moved from the cytoplasm toward the apical side (Fig 8C^1^–8F^3^, S12 and S13 Movies). In the late stage 13 egg chamber, when the polar cell protrusion is fully elongated, we observed LAMP1 puncta extruding from the protrusion tip towards the oocyte (Fig 8G^1^–8J^3^, S14 and S15 Movies).

To check if the lysosomal movement was responsible for forming an open micropyle, we first sought to disrupt lysosomal activity in the polar cells and assess its impact on micropyle channel formation. To down-regulate lysosomal activity, we identified several candidate genes that regulate lysosomal function and cause female sterility (S1 Table: Mitf, ATG1, Vha16). Microphthalmia-associated transcription factor (Mitf) emerged as a promising candidate among these. Mitf encodes a b-HLH-Zip transcription factor that regulates lysosomal activity by controlling the expression of vacuolar (H+)-ATPase subunits [54]. Coincidentally, its expression is highly enriched in the egg chamber follicle cells.

To investigate the role of Mitf in lysosomal activity, we overexpressed Mitf RNAi in polar cells using a polar cell-specific *upd-*Gal4 driver. We first examined Fas III, a marker for polar cell protrusions. We found that the downregulation of Mitf did not affect the formation of polar cell protrusions at stage 14 (Fig 8K^1^ and 8L^1^). This suggests that lysosomal activity is not crucial for the initial formation of the protrusions.

Since lysosomal movement occurs after the protrusions are formed, we next examined the micropyle structure using DIC microscopy at high magnification (100×) in Mitf-depleted backgrounds. Interestingly, while polar cell protrusions normally formed in Mitf RNAi egg chambers, the endochorionic canal of the micropyle also developed as expected. However, we observed that the opening of the inner vitelline membrane was significantly affected in the Mitf RNAi background compared to controls (Fig 8K^2^ and 8L^2^). Specifically, a higher percentage of egg chambers showed a opened vitelline membrane in the Mitf RNAi condition (47.3%, *n* = 450 egg chambers) compared to the control group (97.2%, *n* = 550 egg chambers) **(*upd*-Gal4; UAS *dicer II-*97.22% ± 1.29 SEM**, ***n* = 550 egg chambers and *upd*-Gal4; UAS *mitf RNAi-*53.99% ± 4.31 SEM, *n* = 450 egg chambers,** Fig 8M, S6 Data). Additionally, we also downregulated the function of Vacuolar H[^+^] ATPase 16kD (*Vha16)* in the polar cells. Vha16 encodes the proteolipid component, and its associated vascular acidification [54,55]. Similar defects were observed when we disrupted the function of *Vha16*, further supporting the notion that lysosomal activity plays a role in micropyle channel formation and fertilization (*upd*-Gal4; UAS *vha16*^*RNAi*^-50.4%, *n* = 500 egg chambers, *upd*-Gal4; UAS *dicer II*-93.7%, *n* = 450 egg chambers) **(*upd*-Gal4; UAS *dicer II-*93.78% ± 3.21 SEM**, ***n* = 450 egg chambers and *upd*-Gal4; UAS *vha-16 RNAi-*50.44% ± 3.96 SEM, *n* = 500 egg chambers,** S10B–S10D Fig, S13 Data).

To determine whether the opening of the vitelline membrane in the micropyle is essential for sperm entry during fertilization, we conducted a hatchability test using F1 females of the *upd-*Gal4; *UAS-Mitf RNAi* genotype, which were crossed with *w1118* males. The hatchability of embryos was then assessed. Interestingly, we observed that only 33.9% of the embryos hatched in this background (*n* = 130 embryos), compared to the control group 88.9% hatched (*n* = 350 embryos) **(*upd*-Gal4; UAS *dicer II-*88.96% ± 1.38 SEM**, ***n* = 350 embryos and *upd*-Gal4; UAS *mitf RNAi-*33.95% ± 1.92 SEM, *n* = 130 embryos,** Fig 8N, S6 Data). Further to address that, do BCs and Centripetal cells also contribute to lysosome delivery, we downregulated *mitf* function independently in the BCs and Centripetal cells and evaluated the open status of Vitelline Membrane. Since downregulation of *mitf* function either in BCs or the Centripetal cells didn’t impede the opening of the Vitelline Membrane **(*109c1*-Gal4; UAS *dicer II-*94.03% ± 1.07 SEM**, ***n* = 255 egg chambers and *109c1*-Gal4; UAS *mitf RNAi-*93.68% ± 1.82 SEM, *n* = 252 egg chambers and *c289b11*-Gal4; UAS *dicer II-*92.57% ± 1.28 SEM**, ***n* = 243 egg chambers and *c289b11*-Gal4; UAS *mitf RNAi-*93.91% ± 2.19 SEM, *n* = 175 egg chambers,** S10F–S10J Fig, S13 Data), we believe that the lysosomal activity neither in the BCs or the Centripetal cells contribute directly to the opening of Vitelline Membrane. We also conducted the same experiment in a *vha16* knockdown background. In this background, we observed that 52.4% of the eggs hatched (*n* = 425 embryos), compared to 88.8% in the control group (*n* = 332 embryos) **(*upd*-Gal4; UAS *dicer II-*88.9% ± 2.62 SEM**, ***n* = 332 embryos and *upd*-Gal4; UAS *vha-16 RNAi-*48.22% ± 5.47 SEM, *n* = 425 embryos,** S10E Fig, S13 Data), suggesting that the opening of the vitelline membrane in the micropyle is crucial for successful fertilization.

Overall, our data suggest that nanotube-mediated communication between polar cells and the micropyle facilitates micropyle channel formation. This process is critically dependent on lysosomal activity for the proper opening of the vitelline membrane, which is necessary for sperm entry during fertilization.

### JNK signaling in polar cells orchestrates the directional trafficking of lysosomes through the nanotube

**Fig 9:**
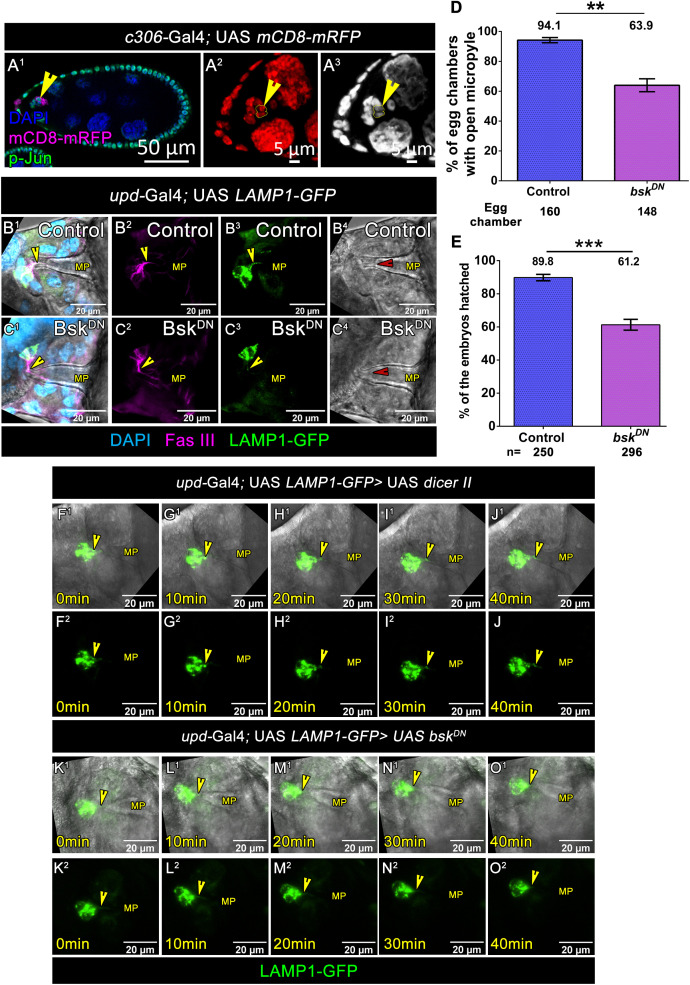
JNK signaling in polar cells orchestrates the directional trafficking of lysosomes through the nanotube. **(A**^**1**^**–A**^**3**^) Stage 9 egg chambers where p-Jun is highly expressed in the outer border cells along with inner polar cells. p-Jun (Green in **A**^**1**^ and gray in **A**^**3**^), DAPI (Blue in **A**^**1**^ and red in **A**^**2**^), mCD8-mRFP (Magenta), yellow arrowheads mark p-Jun expression pattern in the BC cluster, and yellow dotted line represents two PCs. **(B**^**1**^**–B**^**3**^**, C**^**1**^**–C**^**3**^) Comparison of LAMP1-GFP expression pattern in the polar cells at stage 13 egg chambers of indicated genotypes. Fas III (Magenta), LAMP1-GFP (Green), yellow arrowheads are marking LAMP1-GFP puncta in the nanotube. Please note that down regulation of JNK signaling in the polar cells impedes the localization of the LAMP1-GFP puncta into the nanotube. **(B**^**4**^**, C**^**4**^) DIC images of Stage 14 egg chambers of indicated genotypes. Please note that downregulation of Bsk function in the polar cells affects micropyle channel opening, red arrowheads indicate micropyle channel. **(D)** Quantification of % of stage 14 egg chambers with open micropyle. Error bars represent SEM, nonparametric *t* test, **P < 0.01. Detailed quantification in S7 Data. **(E)** Quantification of % of the embryos hatched after 30 hours of egg laying. Error bars represent SEM, nonparametric *t* test, ***P < 0.001. Detailed quantification in S7 Data. **(F**^**1**^**–O**^**2**^) Time-lapse snapshot of lysosomal movements through the nanotube at late stage 13 egg chambers of the indicated genotypes. LAMP1-GFP (Green), yellow arrowheads mark the LAMP1-GFP puncta.

Having established that directed lysosomal trafficking towards the oocyte through nanotubes facilitates Vitelline Membrane opening, we sought to identify the molecular mechanism governing this polarized transport. Previous research has implicated JNK signaling in regulating mitochondrial transport in the axons by functioning as a kinesin–cargo dissociation factor [56,57]. Given our observation that JNK signaling is linked to polar cell protrusion formation, we next examined whether it plays a role in lysosomal transport within the nanotubes. First, we assessed the status of JNK activity by monitoring phospho-Jun (p-Jun), a well-established downstream readout of JNK signaling. In control egg chambers, p-Jun expression was detected in both outer BCs, and in the polar cells (Fig 9A^1^–9A^3^). Though the role of JNK signaling in maintaining the apical–basal polarity of the migrating BC cluster is well-characterized, its function in the polar has largely remained unexplored. To check its role in the polar cells, we overexpressed dominant-negative Bsk construct (Bsk-DN) in the polar cell by *upd*-Gal4 to specifically down-regulate JNK signaling. Unlike our expectation, overexpression of Bsk-DN in the polar cell didn’t impede the nanotube formation. However, the Vitelline membrane was intact and no conspicuous opening could be identified under the microscope. Quantitative analysis revealed that only 63.9% of JNK-depleted egg chambers (*n* = 148 egg chambers) displayed an open micropyle, compared to 94.1% observed in the controls (*n* = 160 egg chambers) **(*upd*-Gal4; UAS *dicer II-*94.18% ± 1.75SEM**, ***n* = 160 egg chambers and *upd*-Gal4; UAS *bsk DN-*63.99% ± 4.31SEM, *n* = 148 egg chambers,** Fig 9B^4^, 9C^4^, 9D, S7 Data). This result corroborated with the hatchability test where 89.8% of embryos (*n* = 250) hatched in the control compared to 61.2% observed in egg chambers with JNK-depleted polar cells (*n* = 296) **(*upd*-Gal4; UAS *dicer II-*89.81% ± 1.90 SEM**, ***n* = 250 egg chambers and *upd*-Gal4; UAS *bsk DN-*61.29% ± 3.27 SEM, *n* = 296 egg chambers,** Fig 9E, S7 Data).

To further check the mechanism, we compared the localization of LAMP1-GFP in control and egg chambers with JNK-depleted polar cell under fixed condition. Unlike the control, where, LAMP1-GFP puncta were readily detected within nanotubes (Fig 9B^1^–9B^3^), JNK-downregulated polar cells exhibited markedly reduced lysosomal entry into nanotubes, with only sporadic puncta observed within these structures (Fig 9C^1^–9C^3^). Live-cell imaging provided dynamic evidence for this trafficking defect. Further time-lapse analysis revealed that LAMP1-GFP puncta failed to undergo directed movement through nanotubes when JNK signaling was suppressed compared to the control (Fig 9F^1^–9O^2^, S16–S19 Movies). Instead, lysosomes accumulated within the polar cell bodies, indicating a specific disruption of the transport machinery rather than lysosome biogenesis.

These findings reveal a previously unrecognized role for JNK signaling in orchestrating directional lysosomal trafficking through cellular nanotubes. Our data demonstrate that JNK-mediated regulation of lysosomal transport is functionally distinct from its role in cell protrusion formation and is specifically required for Vitelline membrane opening at the micropyle. This process represents a critical step in preparing the egg for fertilization, as the micropyle serves as the entry point for sperm. The identification of JNK as a key regulator of this transport process provides new insights into the molecular mechanisms underlying reproductive biology and highlights the importance of polarized organelle trafficking in developmental processes.

## Discussion

**Fig 10:**
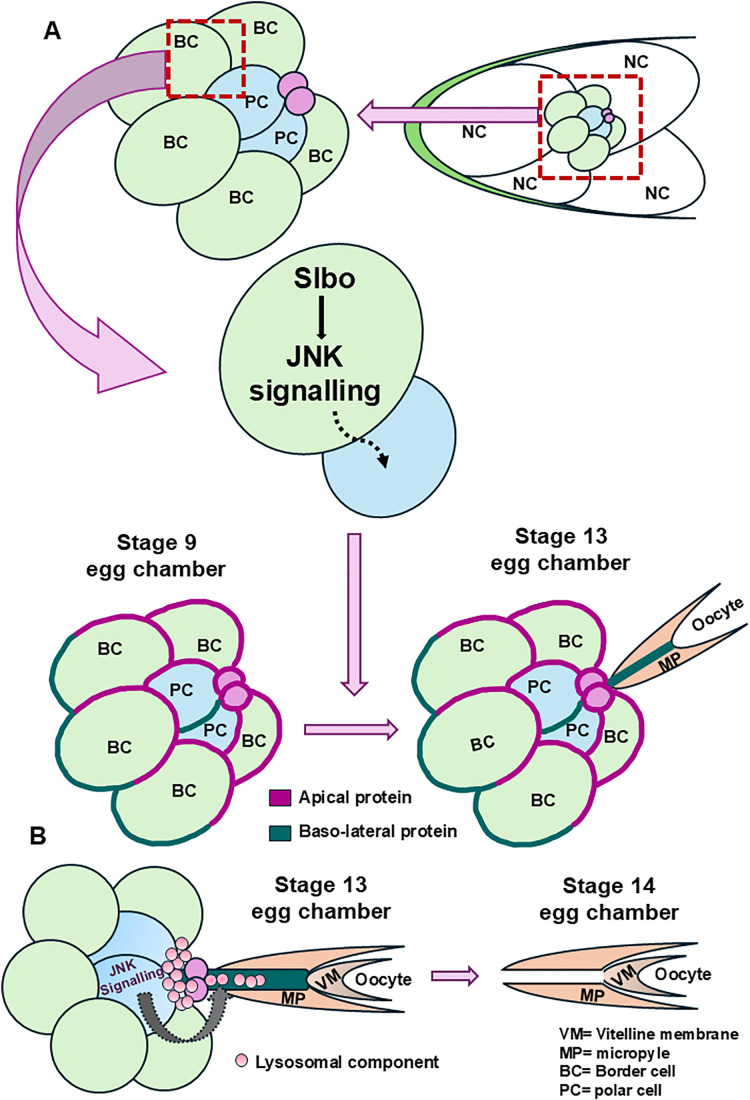
Model. **(A)** Slbo in the outer border cell non- cell autonomously regulates JNK signaling to maintain the proper apical–basal polarity of the migrating border cell cluster. The proper polarity of the border cell cluster facilitates the formation of the polar cell protrusion at stage 13. **(B)** After formation of the polar cell protrusion, JNK signaling in the polar cell regulates the movement of lysosomal components towards the oocyte through the nanotube. The deposition of the lysosomal components at the tip of the polar cell protrusion aids the opening of the vitelline envelope, thus assisting sperm entry during fertilization.

Membrane nanotubes are cytoskeletal protrusions that connect adjacent cells or cells spread over a distance. They primarily function as signaling conduits coordinating metazoan morphogenesis, tumor cell survival, and, occasionally, disease spread. Given the diverse structure and function of membrane nanotubes, there is always an attempt to understand the various roles these structures play in both development and disease. In our study of *Drosophila* micropyle development, we found that a MT-nanotube structure from the specialized anterior polar cells is a prerequisite for creating a narrow channel in the eggshell before fertilization. This channel serves as a conduit for sperm entry during fertilization. We show that Slbo mediated JNK signaling in the outer BC plays an essential role in the maintenance of cluster polarity, which in turn stimulates the MT-nanotube from the central polar cells (Figs 6E^1^–6G, 7F–7I). Strikingly, lysosome function in the polar cells and their transport to the tip of the MT-nanotube plays a crucial role in opening the vitelline layer, rendering the micropyle functional (Figs 8, 9, and S10). Finally, we demonstrate a novel role of JNK signaling in mediating lysosomal transport in the polar cells (Fig 9). Unlike the belief that the BCs just carry the polar cells to the final destination, our data suggests that they also play a critical non-cell autonomous role in forming the polar cell nanotube in late oogenesis by modulating the polarity of the cluster (Fig 10A).

Our data and previous reports indicate that the polar cell protrusion falls under the category of closed MT nanotube. Though the MT nanotube structure has been observed in different systems, their functional output is quite distinct. In the *Drosophila* male germline, the closed MT-nanotube structures assist in retaining the stemness of GSC. In contrast, TNTs are also linked with the trafficking of endosomal/lysosomal vesicles, mitochondria, and autophagosomes as observed in squamous cell carcinoma cells [58]. In this system, we observe that although the polar nanotubes are closed, the transport of the lysosomal machinery is critical for opening the micropyle and role of JNK signaling is unique. We believe this effect is specific as mitochondria tagged with GFP are restricted in the body of the polar cell itself. We observed that the LAMP1-GFP accumulates near the tip, with some vesicles exhibiting back-and-forth movement. Since we didn’t observe the LAMP1-GFP signal fading over time, it suggests against active exocytosis of the lysosomal contents at the nanotube tip. Given the lysosomal activity in the polar cell nanotube is critical for opening the micropyle, one possibility could be a kiss-and-run model where these vesicles partially attach with the nanotube tip, releasing some contents and subsequently are recycled back into the polar cells. Irrespective of how this happens, our genetic data and live cell imaging support that lysosomal function is critical for generating a breach in the rather impervious eggshell (Fig 10B). Our results provide a unique insight as to how the formation of the polar cell nanotube is aided non-cell autonomously by overlying BCs. Our results suggest that the physical attachment of the BCs with polar cells is a prerequisite for segregating the polarity proteins into different domains. This might be crucial for perceiving the signal to initiate nanotube formation and extension. However, unlike the GSC in *Drosophila* testis, where the high concentration of Dpp in the niche cells stimulates MT-nanotube formation, we believe this might not be the case here [59]. The reason for our belief is that polar cell nanotube is formed even in mutants where the BC clusters are far from the micropyle (S6A^1^–S6B^2^, S8E^1^–S8E^3^ Figs). This probably suggests that local buildup of ligands may not be required for polar cell protrusion formation here. Since the polar cell nanotube depends on the stage of oogenesis, we believe there may be some kind of global, systemic signaling, like Ecdysone and Insulin, that can stimulate the process formation. Subsequently, the migrating cluster’s apical region may serve as a specialized region from where the nanotube extension is permissible. We also observed that the nanotube is always directed toward the micropyle, suggesting some chemoattractant signaling may guide the nanotube to the final destination (Fig 1B and 1C). Besides, the chemoattractant, one can’t rule out the presence of local cell–cell or cell–matrix interactions playing a role in giving direction to the polar cell process perse.

In the milieu of new insight into how the migrating BC cluster assists in micropyle morphogenesis, our study opens up several new questions for further examination. It will be worth investigating which signal/s stimulates the nanotube formation and how the microtubular extension is directed toward the apical constriction as it elongates out of the polar cell body. Given that the polar cell nanotube is always directed towards the micropyle, we believe that there must be some signals that guide the polar cell extension. It would also be worth examining the nature of communication between polar cells and micropyle that guides the nanotube to its final destination. As JNK signaling mediates the lysosomal transport in the polar cells and the MT-nanotube, it would be worth examining how this is facilitated at the molecular level. Given that the TNTs are emerging as potential targets for treating tissue injury, combating tumor chemoresistance, and preventing infection spread, understanding the diversity of nanotube structure and function will provide new therapeutic targets and methods to alleviate various disease conditions.

## Material methods

### *Drosophila* stocks

Fly stocks were maintained at 25 °C and crosses were incubated at 25 °C. *upd-*GAL4 (gifted by Dr. Harrison), *slbo*-Gal4, *c289b11*-Gal4 (BL-6981), *109C1*-Gal4 (BL-7020), and *c306*-Gal4 (Bl-3743) were employed for overexpression [60,61]. The following fly lines were used: UAS mCD8-GFP (BL-5237), UAS GFP (BL-6874), UAS Rac-N17 (DGRC 108725 and BL-6292), UAS Spastin (gifted by Dr. Nina Sherwood), UAS Rac2 RNAi (V28926), UAS Tubulin-eGFP (BL-7373), UAS Act 5c GFP (BL-9257), UAS dicer II, UAS pak3 DN [30], UAS Baz- GFP, UAS LAMP1 GFP (Bl-42714), UAS Jra, UAS shg RNAi (Bl-32904), UAS slbo RNAi, *slbo*^*e7b*^(BL-58686), *slbo*^*ry7*^(BL-10740), *puc-*LacZ (BL-98329), *slbo-*LacZ (BL-12227), UAS mitf RNAi (v108519), UAS vha16 RNAi (BL-40923), UAS kayak RNAi, UAS Bsk DN, UASp Shg- GFP (BL-35589), UAS mCD8-mRFP (BL-27399).

### Immunohistochemistry

Two- to 3three-day-old female flies were fattened at 29 °C for 30 hours for all Gal4 based experiments. The remaining were fattened at 25 °C for 30–35 hours. Ovary dissection, fixation, and staining was performed using standard protocol [62]. For immunostaining the following primary antibodies were used: mouse anti-Armadillo monoclonal [N27A1,1:50; DSHB], anti-FAS III monoclonal [7G10,1:50; DSHB], anti-Dlg monoclonal [4F3,1:50; (DSHB)], anti-Coracle monoclonal [C615.16,1:100; (DSHB)], anti-DE-Cadherin monoclonal [DCAD2,1:25; (DSHB)], Rabbit anti GFP polyclonal [A-11122, Invitrogen, 1:2000], Rabbit anti-ß Gal [Invitrogen 1:500], Rabbit anti-Patj (gifted by Elisabeth Knust), Mouse anti p-Jun [KM-1, Santa Cruz Biotechnology, 1:50]. Ovaries were dissected in Schneider’s S2 media, fixed with 4% PFA (158127; Sigma), and blocked using PBT solution {5% Bovine Serum Albumin (BSA), Amresco, catalog no. 0332),0.1% Triton X-100 (Affymetrix, catalog no. T1001) in PBS (P3813; Sigma)}. Resuspended ovaries were then incubated with primary antibody overnight at 4 °C. Secondary antibodies conjugated to Alexa Fluor 488, Alexa Fluor 568, Alexa Fluor 594 (molecular probes, Life Technologies), Secondary antibodies conjugated to Star Red (Mouse- Abberior STRED-1001, Rabbit- Abberior STRED-1002) and Star orange(Mouse- Abberior STORANGE -1001, Rabbit- Abberior STORANGE-1002) were used at a dilution of 1:250 and 1:400, respectively.

For Acetylated Tubulin staining, individual egg chambers were dissected in 1X PEM buffer (60 mM PIPES, 25 mM HEPES, 10 mM EGTA, 4 mM MgSO_4_, pH 6.8) and fixed with 10% formaldehyde in the presence of 1× BRB80 buffer and 1% Tween 20 (Amresco). After fixation, the samples were incubated overnight at 4 °C in 1× PBS containing 1% Triton X-100 and 1× BRB80 buffer. Blocking was performed with 1× PBS (Sigma-Aldrich, catalog no. P3813) containing 1% Triton X-100 (Affymetrix, catalog no. T1001), 5% BSA (Amresco, catalog no. 0332), and 1× BRB80 buffer for 4 hours at room temperature. Mouse anti-acetylated tubulin (Sigma, catalog no. T9026) was used at 1:800 dilution. The primary antibody incubation was carried out overnight at 4 °C. Following this, the samples were washed with 1× PBS containing 0.5% Tween 20 and incubated with secondary antibodies anti-mouse conjugated to Abberior Sar Red (Abberior STRED-1001) at a 1:400 dilution [63].

### Microscopy and imaging

Fly handling was performed under a Olympus SZX16 stereomicroscope. For visualization of the immune-stained samples, Olympus IX81 fluorescence microscope was used. Imaging was conducted using a Zeiss Axio Observer 7 with the Apotome.2 module, a Confocal CLSM 710 (Carl Zeiss, Germany) microscope, and the Abberior facility line 3D-STED microscopy.

For time-lapse imaging, Olympus IX83 spinning disk microscope was employed, with 20% laser power, a 40 ms exposure time, and binning set to 2.

### Live imaging and analysis

Flies used for live cell imaging of polar cell protrusion formations were of the genotype *upd*-GAL4; UAS *mCD8-GFP* and *upd*-GAL4; UAS *GFP*. Time–lapse microscopy was performed as stated earlier [64]. Frames were captured every 2 min interval. Culture conditions were kept same for both control and experiments. On an average, each movie lasted for around 3–4 hours.

### Live imaging to track lysosomal movement

For live cell imaging of lysosomal movements through polar cell protrusions, we used flies with the genotype ***upd*-Gal4; UAS-*LAMP1-GFP*; UAS-*mCD8-RFP***. Time-lapse microscopy was performed as described in **Prasad and Montell (2007)** [64,65]. Images were captured at 100X magnification, at 1.5-minute intervals using inverted Ti2 eclipse microscope (Nikon, Japan) with confocal module (Nikon AX model, with NIS elements software) with minimal crosstalk, employing sequential Galvano scanning and a 2.5 A.U. pinhole, along with DIC imaging. On average, each movie lasted approximately 1 hour.

### Treatment of egg chambers with Nocodazole

*Upd*-Gal4; UAS *GFP* egg chambers were dissected in S2 media containing 15% FBS and 0.5 mg/ml Insulin (Sigma, cat. no. I5550). Egg chambers were transferred to a poly D-Lysine (Cat# P7280 Sigma-Aldrich) coated confocal dish, and the existing medium was replaced with Nocodazole (Sigma-Aldrich, M1404) (final concentration 66 or 132 µM) containing media. The egg chambers were imaged under fluorescence microscope at regular intervals. Media was replaced after every 1 hour of imaging. Control egg chambers were treated with an equal amount of DMSO, and imaging was done at regular intervals with media replacement at every 1 hour of imaging. After imaging, the polar cell protrusion was measured using ImageJ software.

### Data quantitation and statistics

#### Measurement of polar cell protrusion length.

The images of polar cell protrusion were analyzed in ImageJ software, and a scale was assigned. Since the images were taken at 40× magnification, the scale of images was changed from pixels to µm with the help of reference images. The region of polar cell extension beyond the surface of the existing polar cell membrane at the tapered end was marked to measure polar cell protrusion length. The protrusion was marked with a freehand line selection tool, and the length was determined using the “Measure” option. Since the shape of the polar cell in late oogenesis is cone-shaped, any extension above 0.2 µm wass considered to be a protrusion. Absolute Polar cell protrusion length was measured at every 2 min time interval and plotted. Δlength was calculated as difference between two successive time points.

#### Quantification of polar cell protrusion formation.

Strictly stage 13/ stage 14 egg chambers were considered for scoring polar cell protrusion formation. Stage 13/ stage 14 egg chambers can be identified by observing the complete structure of the dorsal appendages and removal of nurse cell can represent the stage 14 egg chambers. The egg chambers were marked by the Fas III, which marks the polar cell protrusion. A total number of BC clusters belonging to the individual category was counted, and the percentage protrusion formation calculated using the following formula: % of stage 14 egg chambers with polar cell protrusion = (Number of egg chambers exhibiting polar cell protrusion/Total number of egg chambers) * 100.

#### Quantification of width of the polar cell protrusion.

Egg chambers were stained with Acetylated Tubulin and mounted in anti-fade mounting media. Images of the polar cells were acquired as z-sections at regular intervals using a 60× magnification on the ABBERIOR 3D STED facility line microscopy. Imaging was conducted with a 12% depletion laser power (775 nm wavelength), accumulation set to 3, dual time at 5, a pixel size of 30 nm, and a pinhole of 1 AU.

To focus on the protrusion of the polar cell, we zoomed in and took additional z-sections at regular intervals with the same 60× magnification. The imaging parameters were set with accumulation at 3, dual time at 5, and a finer pixel size of 15 nm.

The images of the polar cell protrusion were processed using ImageJ software, where a scale bar was applied based on reference images. Since the images were taken at 60× magnification, the scale was converted from pixels to micrometres. The region of the polar cell that extended beyond the surface of the existing membrane was identified and marked using the freehand selection tool. The width of the protrusion was then measured at various positions, from the base to the tip of the protrusion, using the “Measure” function in ImageJ.

#### *puc*-LacZ intensity quantification.

*puc-*LacZ contains a regulatory sequence from the *puckered (puc)* gene that drives the expression of the *lacZ* reporter gene, reflecting the transcriptional activity of the *puc* locus. However, this construct does not provide information on the translation or production of Puckered protein.

To assess *lacZ* levels, immunostaining was performed using a primary antibody against the β-Gal protein. The BC clusters from egg chambers were imaged at 40× magnification, capturing z-sections at regular intervals with consistent exposure settings for both experimental and control samples. The z-stack images were merged to generate a 2D maximum intensity projection (MIP) image. The individual BCs were outlined in the MIP image, and the mean lacZ intensity was quantified using Zen 2012 software. The values were then normalized to the DAPI intensity of the corresponding cells. The average intensity of the control samples was set to 1, and the experimental intensities were expressed as fold changes relative to the control. Imaging was performed on a Zeiss Axio Observer 7 equipped with the Apotome.2 module, and data analysis was conducted using Zen 2012 software.

#### Egg hatchability assay.

Virgin F1 females from the crosses of the *upd*-Gal4 with UAS *mitf RNAi*, UAS *vha16 RNAi*, UAS *bsk-DN*, and UAS *dicer II* (used as a control) were collected. These females were then crossed with *w1118* males and incubated at 29 °C for Gal4 activation. After 3 days, the cages with these flies were set up and kept at 29 °C for an additional 1 day. The following day, embryos were collected for 2 hours and then incubated at 25 °C for a minimum of 30 hours. After incubation, embryos hatching was evaluated. The percentage of embryos hatched was calculated as:


Percentageofembryoshatched=(Numberofeggshatchedafter30hours/Totalnumberofeggs laid)*100.

#### Details of statistical analysis.

The difference in means was determined using a two-tailed *t* test with unequal variance in GraphPad Prism 6.0. All error bars represent the Standard Error of the Mean (SEM). Statistical significance is indicated on the figures using the following scale:

**** for *p*-value <0.0001, *** for *p*-value <0.001, ** for *p*-value <0.01, * for 0.01 < *p* < 0.05, ns for *p* > 0.05 (not significant).

## Supporting information

S1 Fig Expression pattern of different Gal4 driver in the oogenesis. **(A–C)** Expression pattern of *slbo-*Gal4 at different developmental stages of the egg chamber. *slbo-*Gal4 mainly expressed in the outer border cells, centripetal cells, anterior follicle cells and some posterior follicle cells. mCD8-GFP (Green) and DAPI (Red), lower white inset indicates BC cluster. **(D–F)** Expression pattern of *109C1-*Gal4 at different developmental stages of the egg chamber. *109C1-*Gal4 specifically expressed in the outer border cells. mCD8-GFP (Green) and DAPI (Red), lower white inset indicates BC cluster. **(G–I)** Expression pattern of *upd-*Gal4 at different developmental stages of the egg chamber. *upd-*Gal4 specifically expressed in the polar cells. mCD8-GFP (Green) and DAPI (Red), lower white inset indicates BC cluster. **(J–L)** Expression pattern of *c306-*Gal4 at different developmental stages of the egg chamber. *c306-*Gal4 mainly expressed in the border cells, polar cells, anterior follicle cells. mCD8-GFP (Green) and DAPI (Red), lower white inset indicates BC cluster. **(M, N)** Expression pattern of *c289b11-*Gal4 at different developmental stages of the egg chamber. *c289b11-*Gal4 specifically expressed in the centripetal cells. mCD8-GFP (Green) and DAPI (Red), lower white inset indicates BC cluster.
(TIFF)

S2 Fig Polarity marker protein distribution in polar cell at late oogenesis. **(A–C**^**2**^) Distribution of Armadillo in stage 13 egg chamber of indicated genotype. Armadillo does not label the polar cell protrusion. Armadillo (Red), DAPI (Blue), and GFP (Green). White arrows indicate polar cell protrusion. **(D–F**^**2**^) Distribution of Patj in stage 13 egg chamber of indicated genotype. Patj does not label the polar cell protrusion. Patj (Red), DAPI (Blue), and GFP (Green). White arrows indicate polar cell protrusion. **(G–I**^**2**^) Distribution of Dlg in stage 13 egg chamber of indicated genotype. Dlg does not label the polar cell protrusion. Dlg (Red), DAPI (Blue), and GFP (Green). White arrows indicate polar cell protrusion. **(J–L**^**2**^) Distribution of Coracle in stage 13 egg chamber of indicated genotype. Coracle is labeled in the polar cell protrusion. Coracle (Red), DAPI (Blue), and GFP (Green). White arrows indicate polar cell protrusion. **(M–O**^**2**^) Distribution of Fas III in stage 13 egg chambers of indicated egg chambers. Fas III is labeled in the polar cell protrusion. Fas III (Red), DAPI (Blue), and GFP (Green). White arrowheads indicate polar cell protrusion.
(TIFF)

S3 Fig Disruption of Actin does not affect polar cell protrusion formation. **(A**^**1**^**–A**^**2**^) Overexpression of *Act 5C-GFP* in the polar cell labeled the polar cell protrusion. Actin-eGFP (Green), DAPI (Blue). White arrows indicate polar cell protrusion. **(B**^**1**^**–B**^**2**^) Overexpression of *Tub 84B-GFP* in the polar cell labeled the polar cell protrusion. Tubulin-eGFP (Green), DAPI (Blue). White arrows indicate polar cell protrusion. **(C**^**1**^**–E**^**4**^) Time-lapse snapshot of stage 12 egg chambers of the indicated genotypes. **(C**^**1**^**–C**^**4**^) Time-lapse snapshot of Control stage 12 egg chamber of the indicated genotype. **(D**^**1**^**–D**^**4**^) Time-lapse snapshot of Rac1^N17^ overexpressed stage 12 egg chamber of the indicated genotype. **(E**^**1**^**–E**^**4**^) Time-lapse snapshot of Rac2^RNAi^ overexpressed stage 12 egg chamber of the indicated genotype. GFP (Green), Yellow arrowheads mark polar cell. **(F, G)** Quantitative analysis of the length of the polar cell protrusion at each time points. Error bars represent SEM. Detailed quantification in S8 Data.
(TIFF)

S4 Fig Disruption of Actin does not affect polar cell protrusion formation. **(A**^**1**^**–D)** Downregulation of Diaphanous and Enabled function in the polar cells does not impede polar cell protrusion formation. **(A**^**1**^**–C**^**2**^) Stage 13 egg chamber of indicated genotypes, Fas III (Magenta) and DAPI (Cyan). Yellow arrows indicate polar cell protrusion. **(D)** Quantification of % of stage 14 egg chambers with polar cell protrusion. Error bars represent SEM, nonparametric *t* test, ns *P > 0.05*. Detailed quantification in S9 Data.
(TIFF)

S5 Fig Overexpression of Spastin alone in polar cell does not affect polar cell protrusion formation. **(A**^**1**^**–B**^**4**^) Time-lapse snapshot of stage 12 egg chambers of the indicated genotypes. **(A**^**1**^**–A**^**4**^) Time-lapse snapshot of Control stage 12 egg chamber of the indicated genotype. **(B**^**1**^**–B**^**4**^) Time-lapse snapshot of Spastin overexpressed stage 12 egg chamber of the indicated genotype. GFP (Green), Yellow arrowheads mark polar cell. **(C)** Quantitative analysis of the length of the polar cell protrusion at each time points. Error bars represent SEM. Detailed quantification in S10 Data.
(TIFF)

S6 Fig Physical connection between polar cell and the micropyle does not affect polar cell protrusion formation. **(A**^**1**^**–B**^**2**^) Downregulation of Zpg function in the nurse cells affects physical connection between border cell cluster and micropyle but does not affect polar cell process formation. **(A**^**1**^**–B**^**2**^) Stage 13 egg chambers of indicated genotypes, Fas III (Magenta), and DAPI (Cyan). Yellow arrowheads indicate polar cell protrusion.
(TIFF)

S7 Fig Downregulation of Slbo in the polar cell and the centripetal cell does not affect polar cell protrusion formation. **(A**^**1**^**–C)** Downregulation of Slbo function in the outer border cells affects polar cell protrusion formation. **(A**^**1**^**–B**^**2**^) Stage 13 egg chamber of indicated genotypes, Fas III (Magenta) and DAPI (Cyan). White arrows indicate polar cell protrusion. **(C)** Quantification of % of stage 14 egg chambers with polar cell protrusion. Error bars represent SEM, nonparametric *t* test, ****P < 0.0001. Detailed quantification in S11 Data. **(D**^**1**^**–F)** Downregulation of Slbo function in the polar cells does not impede polar cell protrusion formation. **(D**^**1**^**–E**^**2**^) Stage 13 egg chamber of indicated genotypes, Fas III (Magenta) and DAPI (Cyan). Yellow arrows indicate polar cell protrusion. **(F)** Quantification of % of stage 14 egg chambers with polar cell protrusion. Error bars represent SEM, nonparametric *t* test, ns *P > 0.05*. Detailed quantification in S11 Data. **(G**^**1**^**–I)** Downregulation of Slbo function in the centripetal cells does not impede polar cell protrusion formation. **(G**^**1**^**–H**^**2**^) Stage 13 egg chamber of indicated genotypes, Fas III (Magenta) and DAPI (Cyan). Yellow arrows indicate polar cell protrusion. **(I)** Quantification of % of stage 14 egg chambers with polar cell protrusion. Error bars represent SEM, nonparametric t tes*t*, ns *P > 0.05*. Detailed quantification in S11 Data. **(J)** Schematic of polar cell protrusion formation at stage 13. Where light green cells are outer border cells, yellow cells are polar cells, dark green cells are centripetal cells, purple color represents oocyte. Please note that downregulation of *slbo in* the outer border cells impede formation of the polar cell protrusion.
(TIFF)

S8 Fig Downregulation of Pak3 in the border cell affects the polarity of migratory border cell cluster. **(A**^**1**^**–B**^**1**^) Downregulation of Shg function in the border cell affects the distribution of DE-Cadherin localization in the migratory cluster. The DE-Cadherin is localized in the PC-PC, PC-BC, BC-BC junction **(A**^**1**^**, A**^**2**^), which is completely reduced in between BC-BC and BC-PC in the Shg-depleted border cell cluster **(B**^**1**^**, B**^**2**^). DE-Cadherin (Magenta in **A**^**1**^**, B**^**1**^ and Black in **A**^**2**^**, B**^**2**^), DAPI (Cyan) inset box represents reference egg chamber of indicated genotypes, red line indicates the outline of BC cluster. **(C, D)** Downregulation of Pak3 function in the border cell affects the distribution of Armadillo localization in the migratory cluster. The Armadillo is localized in the PC-PC, PC-BC, BC-BC junction **(C)**, which is altered in the Pak3-depleted border cell cluster **(D),** Armadillo (Black), red line indicates the outline of BC cluster, inset box represents reference egg chamber of indicated genotypes, showing Armadillo (Magenta) and DAPI (Cyan). **(E**^**1**^**–E**^**3**^) Stage 13 egg chamber of indicated genotype, Fas III (Magenta), and DAPI (Cyan), yellow arrows indicate polar cell protrusion.
(TIFF)

S9 Fig Downregulation of JNK signaling in the migratory border cells affects polar cell protrusion formation. **(A, B)** Downregulation of Slbo function in the border cell affects the distribution of DE-Cadherin localization in the migratory cluster. The DE-Cadherin is localized in the PC-PC, PC-BC, BC-BC junctions **(A)**, which is altered in Slbo-depleted border cell cluster **(B)**, DE-Cadherin (Black), red line indicates the outline of BC cluster, inset box represents reference egg chamber of indicated genotypes, showing DE-Cadherin (Magenta) and DAPI (Cyan). **(C–E)** Overexpression of Jra in slbo mutant border cells rescues the polarity of the border cell cluster. The DE-Cadherin localization is altered in the *slbo* mutant border cell cluster **(D),** and it rescues in overexpression of Jra in *slbo* mutant cluster **(E).** DE-Cadherin (Black), red line indicates the outline of BC cluster, inset box represents reference egg chamber of indicated genotypes, showing DE-Cadherin (Magenta) and DAPI (Cyan). **(F**^**1**^**–I)** Downregulation of JNK signaling in the border cells affects polar cell protrusion formation. **(F**^**1**^**–H**^**2**^) Stage 13 egg chamber of indicated genotypes, Fas III (Magenta) and DAPI (Cyan), yellow arrows indicate polar cell protrusion. **(I)** Quantification of % of stage 14 egg chambers with polar cell protrusion. Error bar SEM, nonparametric *t* test, ****P < 0.0001. Detailed quantification in S12 Data.
(TIFF)

S10 Fig Lysosomal component of the Polar cell facilitates the Micropyle channel opening. **(A**^**1**^**–A**^**2**^) The micropyle cone is highly acidic in nature. Stage 14 egg chamber was stained by lysotracker, where lysotracker in white, F-Actin in green, and DAPI in blue, the yellow arrows indicate the micropyle cone structure. **(B–D)** Downregulation of Vha-16 function in the polar cells affects micropyle channel opening. **(B, C)** DIC images of Stage 14 egg chambers of indicated genotypes, yellow arrows indicate micropyle channel, and white arrows indicate the micropyle cone. **(D)** Quantification of % of stage 14 egg chamber with open micropyle. Error bars represent SEM, nonparametric *t* test, **P < 0.01. Detailed quantification in S13 Data. **(E)** Quantification of % of embryos hatched after 30 hours of egg laying. Error bars represent SEM, nonparametric *t* test, ***P < 0.001. Detailed quantification in S13 Data. **(F–J)** Downregulation of Mitf function in the BCs and CFCs does not impede micropyle channel opening. **(F–I)** DIC images of Stage 14 egg chamber of indicated genotypes, yellow arrows indicate micropyle channel, and white arrows indicate the micropyle cone. **(J)** Quantification of % of stage 14 egg chambers with open micropyle. Error bars represent SEM, nonparametric *t* test, ns *P > 0.05*. Detailed quantification in S13 Data.
(TIFF)

S1 Table List of Lysosomal proteins. (PDF)

S1 Movie Three-dimensional 2D-STED reconstruction of a stage 14 wild type polar cell protrusion (in left, X-axis rotation and in right Y-axis rotation view) marked with Baz-GFP (Green), Fas III (Magenta), and DAPI (Blue). Where Baz-GFP marked the apical constriction and Fas III labeled the polar ell protrusion.
(MP4)

S2 Movie Time-lapse movie of a stage 12 egg chamber of the *upd*-Gal4;UAS *mCD8-GFP*, showing polar cell protrusion formation. mCD8-GFP is expressed specifically in the Polar cell and labels the cellular protrusion. Scale bar is shown at the bottom right. Speed of the movies 5 Frame Per Second (FPS) and total duration of the movies is 5 hours.
(MP4)

S3 Movie Time-lapse movie of a stage 12 egg chamber of the *upd*-Gal4; UAS *GFP*/UAS *GFP*, showing normal polar cell protrusion formation. GFP is expressed specifically in the Polar cell and labels the cellular protrusion. Scale bar is shown at the bottom right. Speed of the movies 5 FPS and total duration of the movies is 2 hours.
(MP4)

S4 Movie Time-lapse movie of a stage 12 egg chamber of the *upd*-Gal4; UAS *GFP*;UAS *rac1*^*N17*^, showing normal polar cell protrusion formation. GFP is expressed specifically in the Polar cell and labels the cellular protrusion. Scale bar is shown at the bottom right. Speed of the movies 5 FPS and total duration of the movies is 2 hours.
(MP4)

S5 Movie Time-lapse movie of a stage 12 egg chamber of the *upd*-Gal4;UAS *GFP*;UAS *rac2*^*RNAi*^, showing normal polar cell protrusion formation. GFP is expressed specifically in the Polar cell and labels the cellular protrusion. Scale bar is shown at the bottom right. Speed of the movies 5 FPS and total duration of the movies is 2 hours.
(MP4)

S6 Movie Time-lapse movie of a stage 12 egg chamber of the *upd*-Gal4;UAS *GFP*/UAS *GFP* treated with DMSO, showing polar cell protrusion formation. GFP is expressed specifically in the Polar cell and labels the cellular protrusion. Scale bar is shown at the bottom right. Speed of the movies 5 FPS and total duration of the movies is 2 hours.
(MP4)

S7 Movie Time-lapse movie of a stage 12 egg chamber of the *upd*-Gal4;UAS *GFP*/UAS *GFP* treated with 66 µM nocodazole, showing normal polar cell protrusion formation. GFP is expressed specifically in the Polar cell and labels the cellular protrusion. Scale bar is shown at the bottom right. Speed of the movies 5 FPS and total duration of the movies is 2 hours.
(MP4)

S8 Movie Time-lapse movie of a stage 12 egg chamber of the *upd*-Gal4; UAS *GFP*/UAS *GFP* treated with 132 µM nocodazole, impeding polar cell protrusion formation. GFP is expressed specifically in the Polar cell and labels the cellular protrusion. Scale bar is shown at the bottom right. Speed of the movies 5 FPS and total duration of the movies is 2 hours.
(MP4)

S9 Movie Time-lapse movie of a stage 12 egg chamber of the *upd*-Gal4; UAS *GFP*/UAS *spastin* treated with 66 µM nocodazole, impeding polar cell protrusion formation. GFP is expressed specifically in the Polar cell and labels the cellular protrusion. Scale bar is shown at the bottom right. Speed of the movies 5 FPS and total duration of the movies is 2 hours.
(MP4)

S10 Movie Time-lapse movie of a stage 12 egg chamber of the *upd*-Gal4;UAS *GFP*/UAS *GFP*, showing normal polar cell protrusion formation. GFP is expressed specifically in the Polar cell and labels the cellular protrusion. Scale bar is shown at the bottom right. Speed of the movies 5 FPS and total duration of the movies is 2 hours.
(MP4)

S11 Movie Time-lapse movie of a stage 12 egg chamber of the *upd*-Gal4; UAS *GFP*/UAS *spastin*, showing normal polar cell protrusion formation. GFP is expressed specifically in the Polar cell and labels the cellular protrusion. Scale bar is shown at the bottom right. Speed of the movies 5 FPS and total duration of the movies is 2 hours.
(MP4)

S12 Movie Time-lapse movie of early stage 13 egg chamber of the *upd*-Gal4; UAS *LAMP1-GFP*; UAS *mCD8-mRFP*, showing LAMP1-GFP movements through polar cell protrusion. LAMP1-GFP is marked by Green and mCD8-mRFP marked by red, which is expressed specifically in the Polar cell. Scale bar is shown at the bottom right. Speed of the movies 10 FPS and total duration of the movies is 1 hour.
(MP4)

S13 Movie Time-lapse movie of early stage 13 egg chamber of the *upd*-Gal4; UAS *LAMP1-GFP*; UAS *mCD8-RFP*, showing LAMP1-GFP movements through polar cell protrusion. LAMP1-GFP is marked by Green, which is expressed specifically in the Polar cell. Scale bar is shown at the bottom right. Speed of the movies 10 FPS and total duration of the movies is 1 hour.
(MP4)

S14 Movie Time-lapse movie of late stage 13 egg chamber of the *upd*-Gal4; UAS *LAMP1-GFP*; UAS *mCD8-mRFP*, showing LAMP1-GFP puncta present in the tip of the polar cell protrusion. LAMP1-GFP is marked by Green and mCD8-mRFP marked by red, which is expressed specifically in the Polar cell. Scale bar is shown at the bottom right. Speed of the movies 10 FPS and total duration of the movies is 1 hour.
(MP4)

S15 Movie Time-lapse movie of late stage 13 egg chamber of the *upd*-Gal4; UAS *LAMP1-GFP*; UAS *mCD8-mRFP*, showing LAMP1-GFP movements through polar cell protrusion. LAMP1-GFP is marked by Green, which is expressed specifically in the Polar cell. Scale bar is shown at the bottom right. Speed of the movies 10 FPS and total duration of the movies is 1 hour.
(MP4)

S16 Movie Time-lapse movie of late stage 13 egg chamber of the *upd*-Gal4; UAS *dicer II*/UAS *LAMP1-GFP*, showing LAMP1-GFP movements through polar cell protrusion. LAMP1-GFP is marked by Green, which is expressed specifically in the Polar cell. Scale bar is shown at the bottom right. Speed of the movies 10 FPS and total duration of the movies is 1 hour.
(MP4)

S17 Movie Time-lapse movie of late stage 13 egg chamber of the *upd*-Gal4; UAS *dicer II*/UAS *LAMP1-GFP*, showing LAMP1-GFP movements through polar cell protrusion. LAMP1-GFP is marked by Green, which is expressed specifically in the Polar cell. Scale bar is shown at the bottom right. Speed of the movies 10 FPS and total duration of the movies is 1 hour.
(MP4)

S18 Movie Time-lapse movie of late stage 13 egg chamber of the *upd*-Gal4;UAS *LAMP1-GFP;* UAS *bsk*^*DN*^, showing LAMP1-GFP restrict to the polar cell body and impede the movements of LAMP1 through nanotube. LAMP1-GFP is marked by Green, which is expressed specifically in the Polar cell. Scale bar is shown at the bottom right. Speed of the movies 10 FPS and total duration of the movies is 1 hour.
(MP4)

S19 Movie Time-lapse movie of late stage 13 egg chamber of the *upd*-Gal4;UAS *LAMP1-GFP;* UAS *bsk*^*DN*^, showing LAMP1-GFP restrict to the polar cell body and impede the movements of LAMP1 through nanotube. LAMP1-GFP is marked by Green, which is expressed specifically in the Polar cell. Scale bar is shown at the bottom right. Speed of the movies 10 FPS and total duration of the movies is 1 hour.
(MP4)

S1 Data Polar cell protrusion dynamics. **Panel K:** Length of the polar cell protrusions. **Panel L:** Delta changes of polar cell protrusion length. **Panel N:** Width of the protrusions.
(XLSX)

S2 Data Length of the polar cell protrusion in disrupting tubulin background. **Panel E:** Nocodazole treatment with Spastin over-expression in polar cell impede the length of polar cell protrusion.
(XLSX)

S3 Data Depletion of slbo in BCs impede polar cell protrusion formation. **Panel E:** Slbo mutant impede polar cell protrusion formation. **Panel J:** Slbo knockdown in outer border cell impede polar cell protrusion formation.
(XLSX)

S4 Data Polarity of the border cell cluster regulate polar cell protrusion formation. **Panel D:** Downregulation of Shg in BCs impede polar cell protrusion formation. **Panel G:** Downregulation of Pak3 in BCs impede polar cell protrusion formation.
(XLSX)

S5 Data JNK signaling downstream of slbo regulate polar cell protrusion formation. **Panel E:** Quantification of puc-lacZ intensity in slbo knockdown BCs. **Panel I:** Over-expression of Jra rescues the slbo-depleted phenotype.
(XLSX)

S6 Data Downregulation of lysosomal function in polar cell impede opening of VM. **Panel M:** Downregulation of Mitf in the PCs impede the opening of MP channel at stage 14. **Panel N:** Downregulation of Mitf in the PCs impede hatchability of embryos.
(XLSX)

S7 Data Downregulation of JNK in PCs impede opening of VM. **Panel D:** Over-expression of Bsk DN in the PCs impede the opening of MP channel at stage 14. **Panel E:** Over-expression of Bsk DN in the PCs impede hatchability of embryos.
(XLSX)

S8 Data Disruption of Actin in the PCs does not impede the length of Polar Cell protrusion. **Panel F:** Downregulation of Rac1 in the PCs does not impede the length of polar cell protrusion. **Panel G:** Downregulation of Rac2 in the PCs does not impede the length of polar cell protrusion.
(XLSX)

S9 Data Disruption of Actin in the PCs does not impede the formation of Polar Cell protrusion. **Panel D:** Over-expression of Dia and Ena RNAi in the PCs does not impede the formation of Polar Cell protrusion.
(XLSX)

S10 Data Over-expression of Spastin alone in the PCs does not impede the length of polar cell protrusion formation. **Panel C:** Over-expression of Spastin alone in the PCs does not impede the length of polar cell protrusion formation.
(XLSX)

S11 Data Slbo in outer BCs non-cell autonomously regulate polar cell protrusion formation. **Panel C:** Downregulation of Slbo in BCs impede polar cell protrusion formation. **Panel F:** Downregulation of Slbo in PCs does not impede polar cell protrusion formation. **Panel I:** Downregulation of Slbo in CFCs does not impede polar cell protrusion formation.
(XLSX)

S12 Data Downregulation of JNK signaling in BCs impede polar cell protrusion formation. **Panel I:** Over-expression of Kayak RNAi and Bsk DN in BCs impede polar cell protrusion formation.
(XLSX)

S13 Data Downregulation of lysosomal function in polar cell impede opening of VM. **Panel D:** Downregulation of Vha-16 in the PCs impede the opening of MP channel at stage 14. **Panel E:** Downregulation of Vha-16 in the PCs impede hatchability of embryos. **Panel J:** Downregulation of Mitf in the BCs and the CFCs does not impede opening of VM.
(XLSX)

## Abbreviations

BSA: Bovine Serum Albumin
Dia: Diaphanous
DIC: differential interference contrast
Ena: Enabled
Fas III: Fasciclin III
GSC: germline stem cells
JNK: Jun kinase
LAMP1: lysosome-associated membrane protein 1
MIP: maximum intensity projection
Mitf: microphthalmia-associated transcription factor
MT: microtubule
Pak3: p21 Activated Kinase 3
Slbo: Slow border cells
TNTs: tunneling nanotubes
