# Supplementary figures and images for: Polar cell membrane nanotubes containing microtubules and acidic vesicles render Drosophila eggs fertile
Source: PLoS Biol. 2025 Dec 2;23(12):e3003533. doi: 10.1371/journal.pbio.3003533 (PMC12697985; doi:10.1371/journal.pbio.3003533)

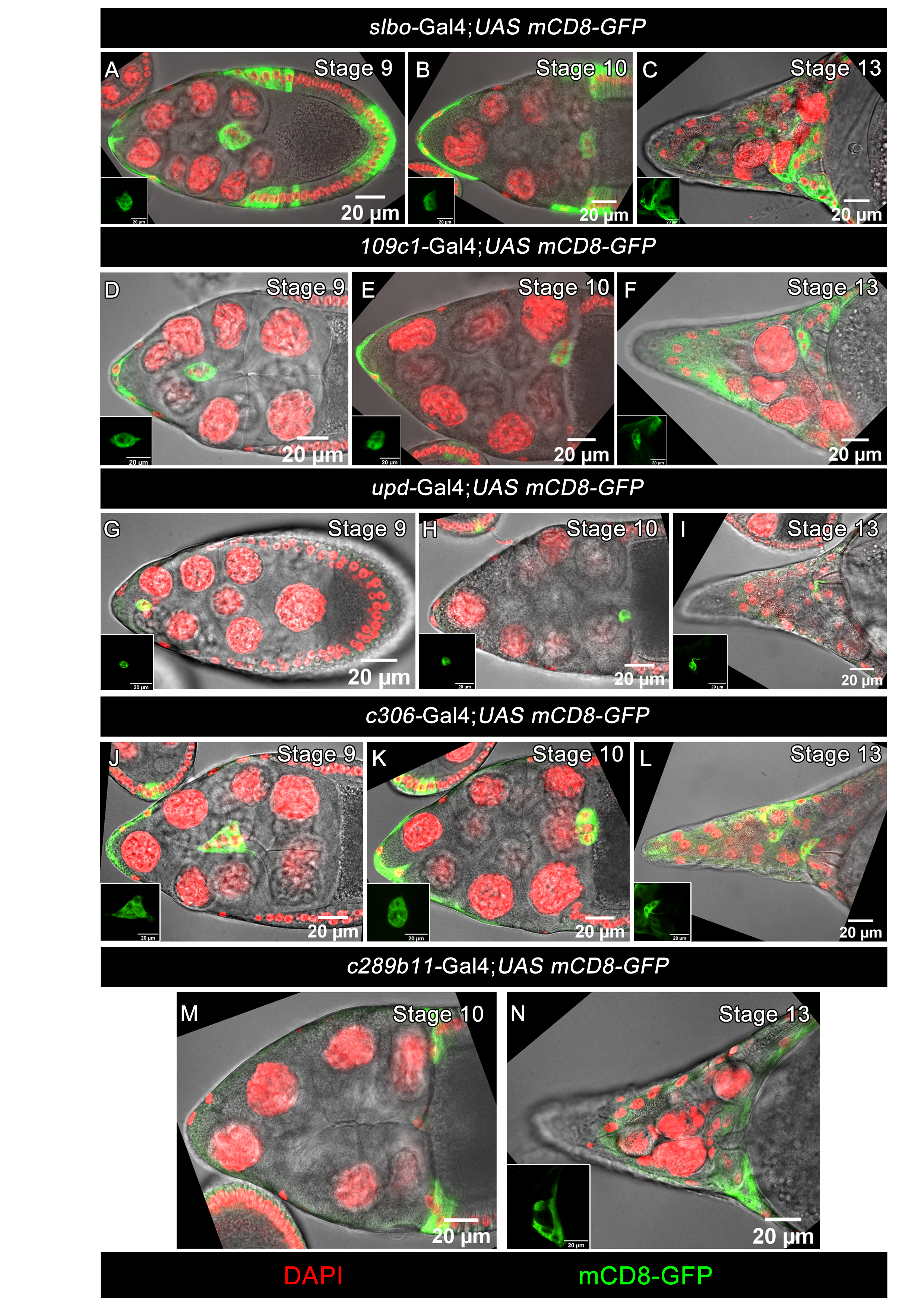

Supplement: S1 Fig — (A–C) Expression pattern of slbo-Gal4 at different developmental stages of the egg chamber. slbo-Gal4 mainly expressed in the outer border cells, centripetal cells, anterior follicle cells and some posterior follicle cells. mCD8-GFP (Green) and DAPI (Red), lower white inset indicates BC cluster. (D–F) Expression pattern of 109C1-Gal4 at different developmental stages of the egg chamber. 109C1-Gal4 specifically expressed in the outer border cells. mCD8-GFP (Green) and DAPI (Red), lower white inset indicates BC cluster. (G–I) Expression pattern of upd-Gal4 at different developmental stages of the egg chamber. upd-Gal4 specifically expressed in the polar cells. mCD8-GFP (Green) and DAPI (Red), lower white inset indicates BC cluster. (J–L) Expression pattern of c306-Gal4 at different developmental stages of the egg chamber. c306-Gal4 mainly expressed in the border cells, polar cells, anterior follicle cells. mCD8-GFP (Green) and DAPI (Red), lower white inset indicates BC cluster. (M, N) Expression pattern of c289b11-Gal4 at different developmental stages of the egg chamber. c289b11-Gal4 specifically expressed in the centripetal cells. mCD8-GFP (Green) and DAPI (Red), lower white inset indicates BC cluster. (TIFF) [file pbio.3003533.s001.tiff]

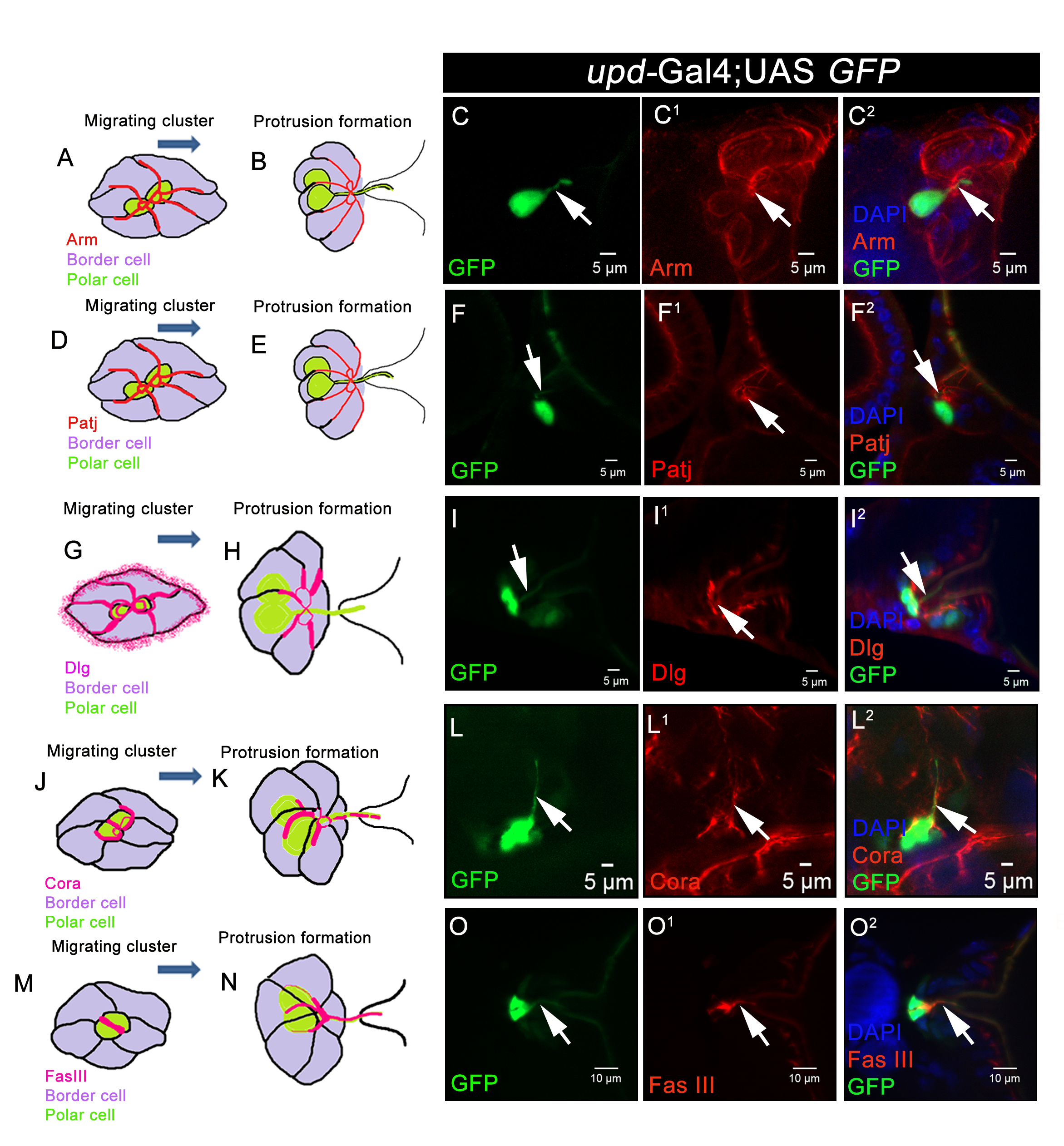

Supplement: S2 Fig — (A–C2) Distribution of Armadillo in stage 13 egg chamber of indicated genotype. Armadillo does not label the polar cell protrusion. Armadillo (Red), DAPI (Blue), and GFP (Green). White arrows indicate polar cell protrusion. (D–F2) Distribution of Patj in stage 13 egg chamber of indicated genotype. Patj does not label the polar cell protrusion. Patj (Red), DAPI (Blue), and GFP (Green). White arrows indicate polar cell protrusion. (G–I2) Distribution of Dlg in stage 13 egg chamber of indicated genotype. Dlg does not label the polar cell protrusion. Dlg (Red), DAPI (Blue), and GFP (Green). White arrows indicate polar cell protrusion. (J–L2) Distribution of Coracle in stage 13 egg chamber of indicated genotype. Coracle is labeled in the polar cell protrusion. Coracle (Red), DAPI (Blue), and GFP (Green). White arrows indicate polar cell protrusion. (M–O2) Distribution of Fas III in stage 13 egg chambers of indicated egg chambers. Fas III is labeled in the polar cell protrusion. Fas III (Red), DAPI (Blue), and GFP (Green). White arrowheads indicate polar cell protrusion. (TIFF) [file pbio.3003533.s002.tiff]

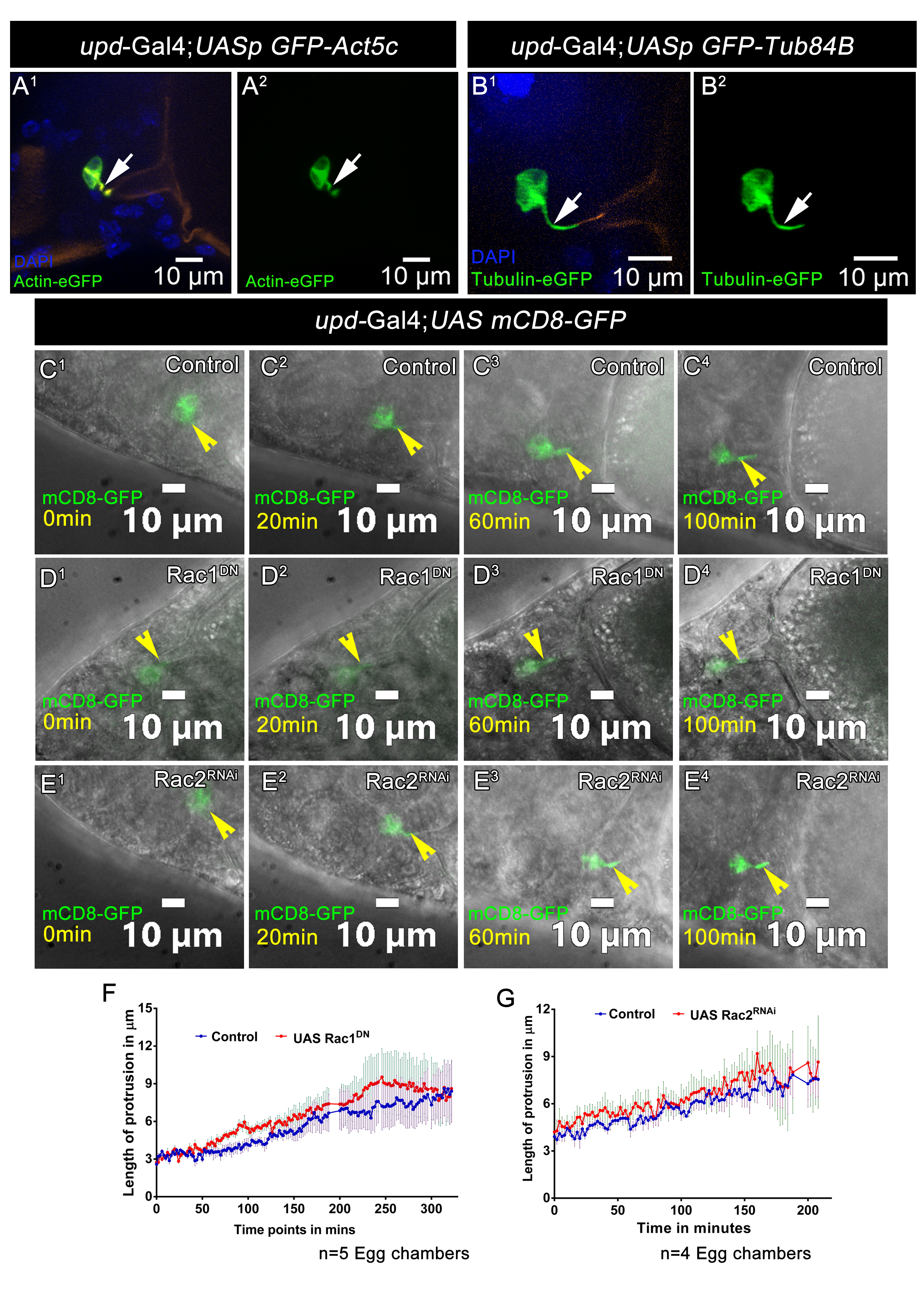

Supplement: S3 Fig — (A1–A2) Overexpression of Act 5C-GFP in the polar cell labeled the polar cell protrusion. Actin-eGFP (Green), DAPI (Blue). White arrows indicate polar cell protrusion. (B1–B2) Overexpression of Tub 84B-GFP in the polar cell labeled the polar cell protrusion. Tubulin-eGFP (Green), DAPI (Blue). White arrows indicate polar cell protrusion. (C1–E4) Time-lapse snapshot of stage 12 egg chambers of the indicated genotypes. (C1–C4) Time-lapse snapshot of Control stage 12 egg chamber of the indicated genotype. (D1–D4) Time-lapse snapshot of Rac1N17 overexpressed stage 12 egg chamber of the indicated genotype. (E1–E4) Time-lapse snapshot of Rac2RNAi overexpressed stage 12 egg chamber of the indicated genotype. GFP (Green), Yellow arrowheads mark polar cell. (F, G) Quantitative analysis of the length of the polar cell protrusion at each time points. Error bars represent SEM. Detailed quantification in S8 Data. (TIFF) [file pbio.3003533.s003.tiff]

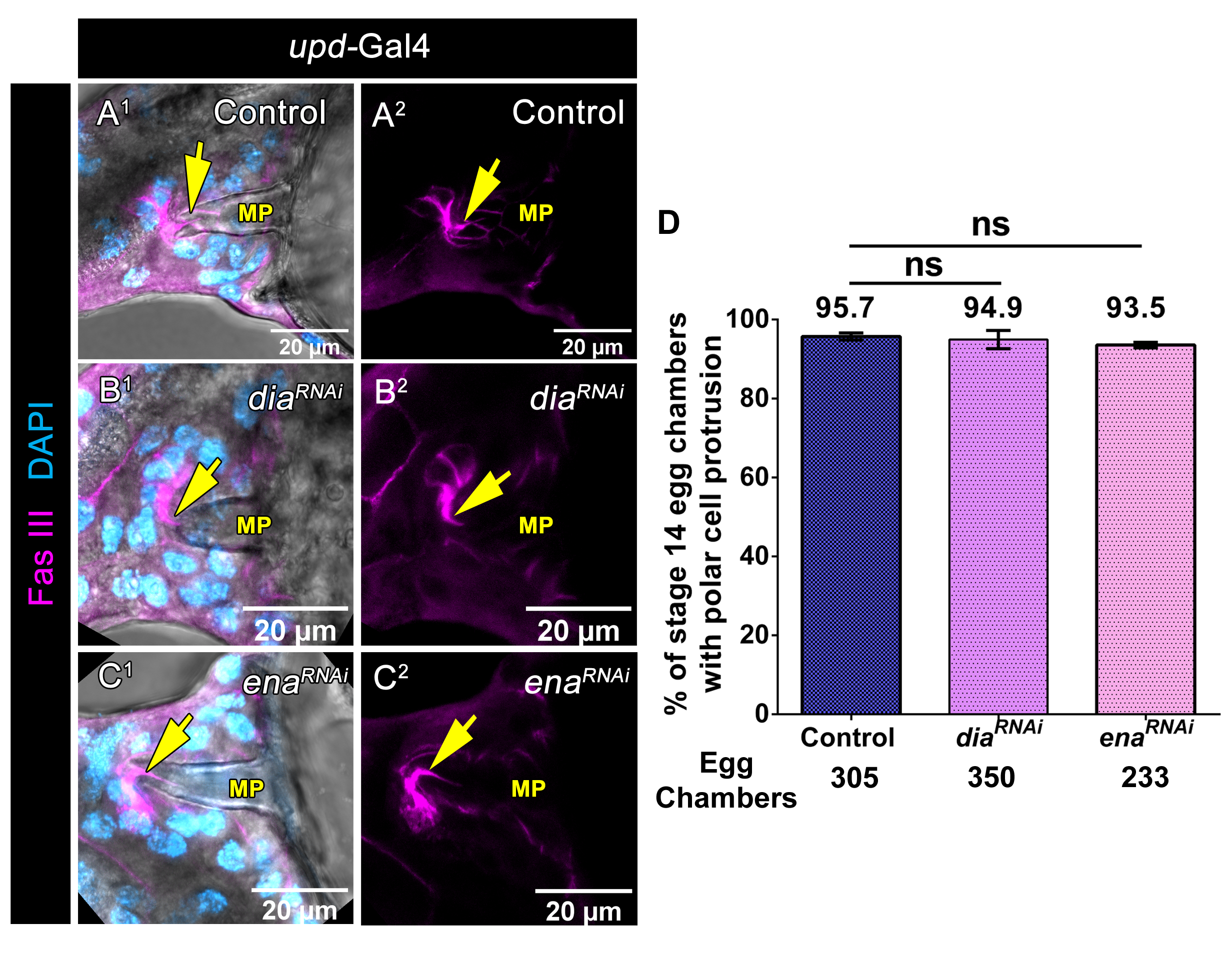

Supplement: S4 Fig — (A1–D) Downregulation of Diaphanous and Enabled function in the polar cells does not impede polar cell protrusion formation. (A1–C2) Stage 13 egg chamber of indicated genotypes, Fas III (Magenta) and DAPI (Cyan). Yellow arrows indicate polar cell protrusion. (D) Quantification of % of stage 14 egg chambers with polar cell protrusion. Error bars represent SEM, nonparametric t test, ns P > 0.05. Detailed quantification in S9 Data. (TIFF) [file pbio.3003533.s004.tiff]

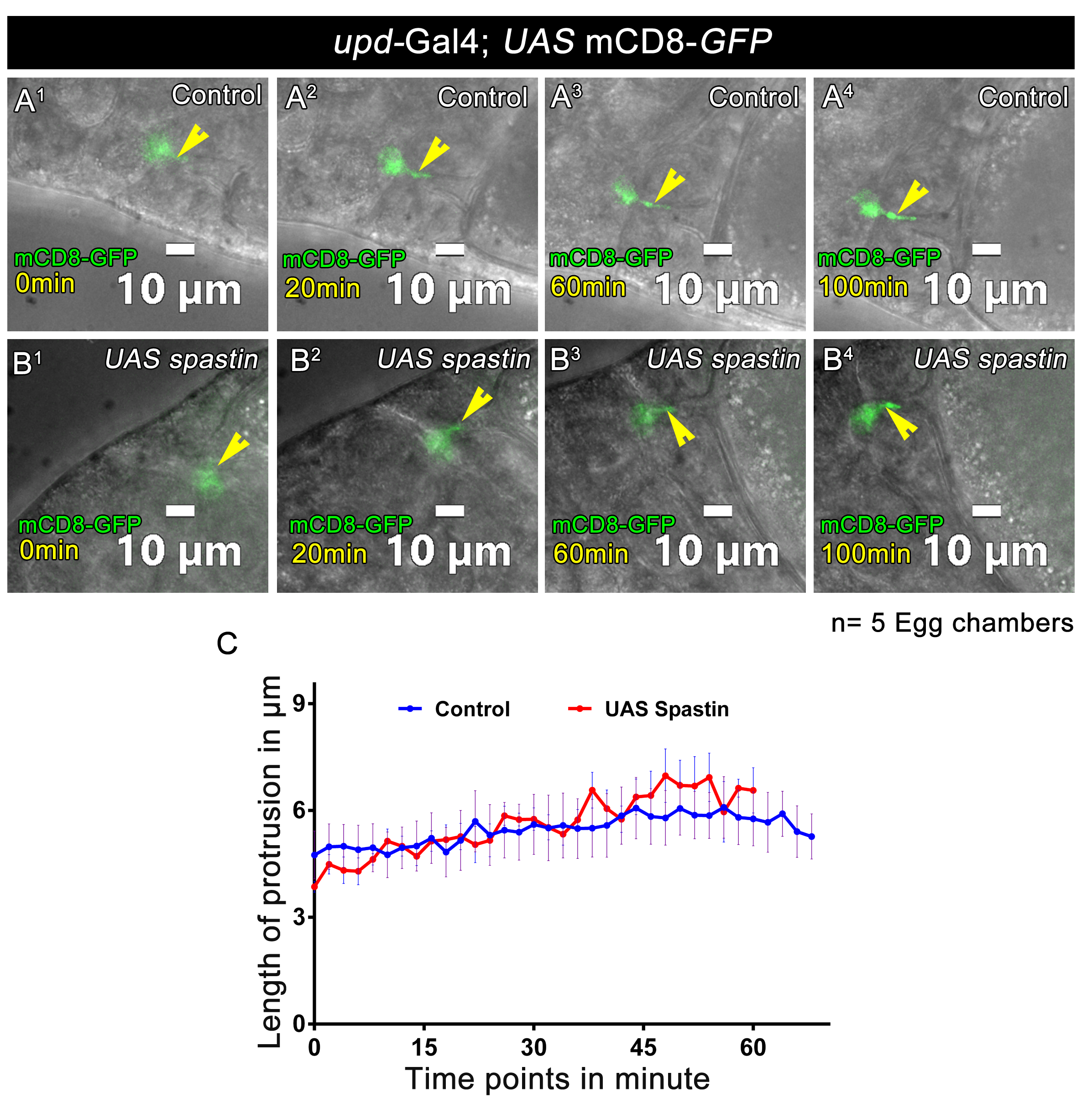

Supplement: S5 Fig — (A1–B4) Time-lapse snapshot of stage 12 egg chambers of the indicated genotypes. (A1–A4) Time-lapse snapshot of Control stage 12 egg chamber of the indicated genotype. (B1–B4) Time-lapse snapshot of Spastin overexpressed stage 12 egg chamber of the indicated genotype. GFP (Green), Yellow arrowheads mark polar cell. (C) Quantitative analysis of the length of the polar cell protrusion at each time points. Error bars represent SEM. Detailed quantification in S10 Data. (TIFF) [file pbio.3003533.s005.tiff]

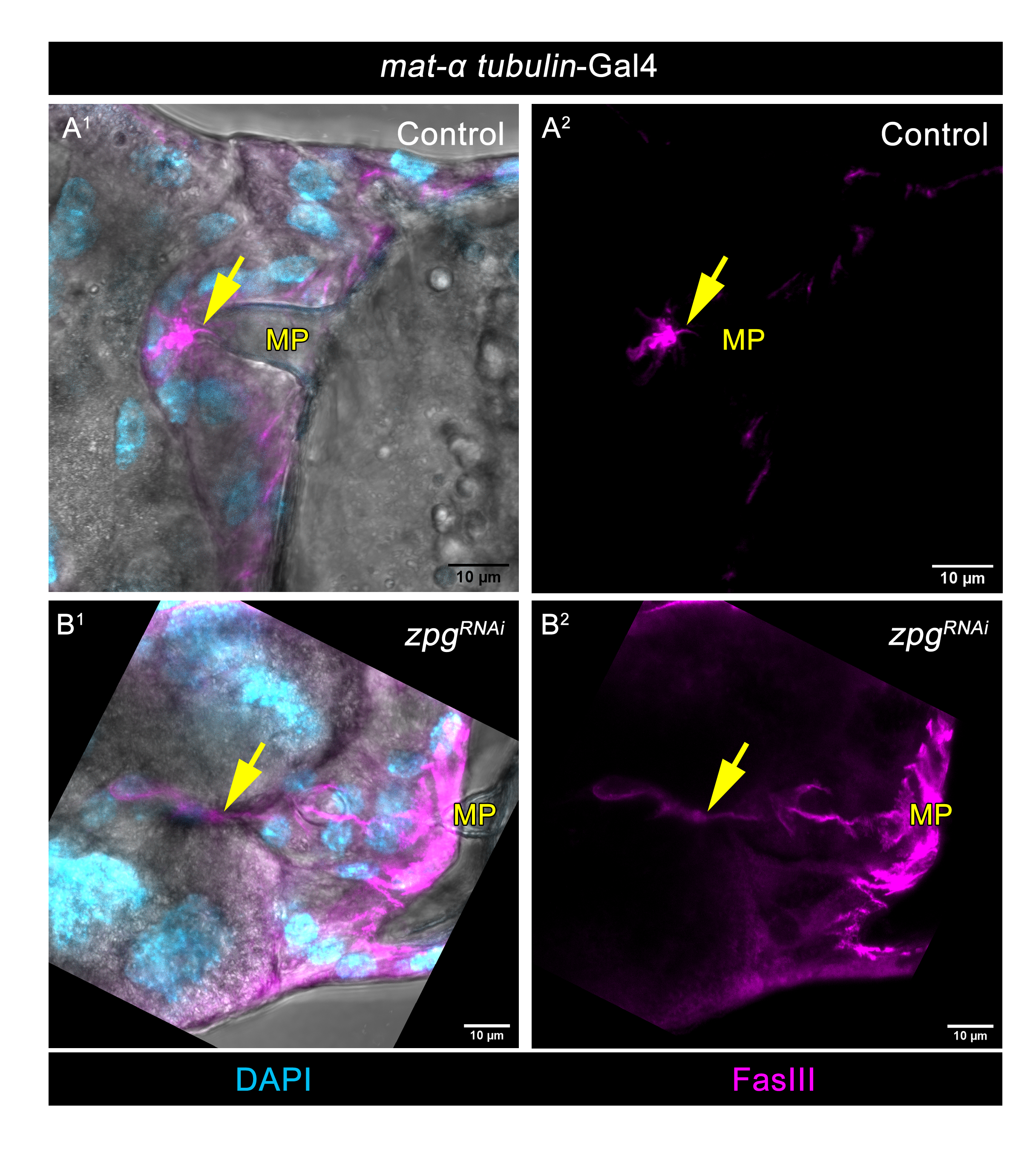

Supplement: S6 Fig — (A1–B2) Downregulation of Zpg function in the nurse cells affects physical connection between border cell cluster and micropyle but does not affect polar cell process formation. (A1–B2) Stage 13 egg chambers of indicated genotypes, Fas III (Magenta), and DAPI (Cyan). Yellow arrowheads indicate polar cell protrusion. (TIFF) [file pbio.3003533.s006.tiff]

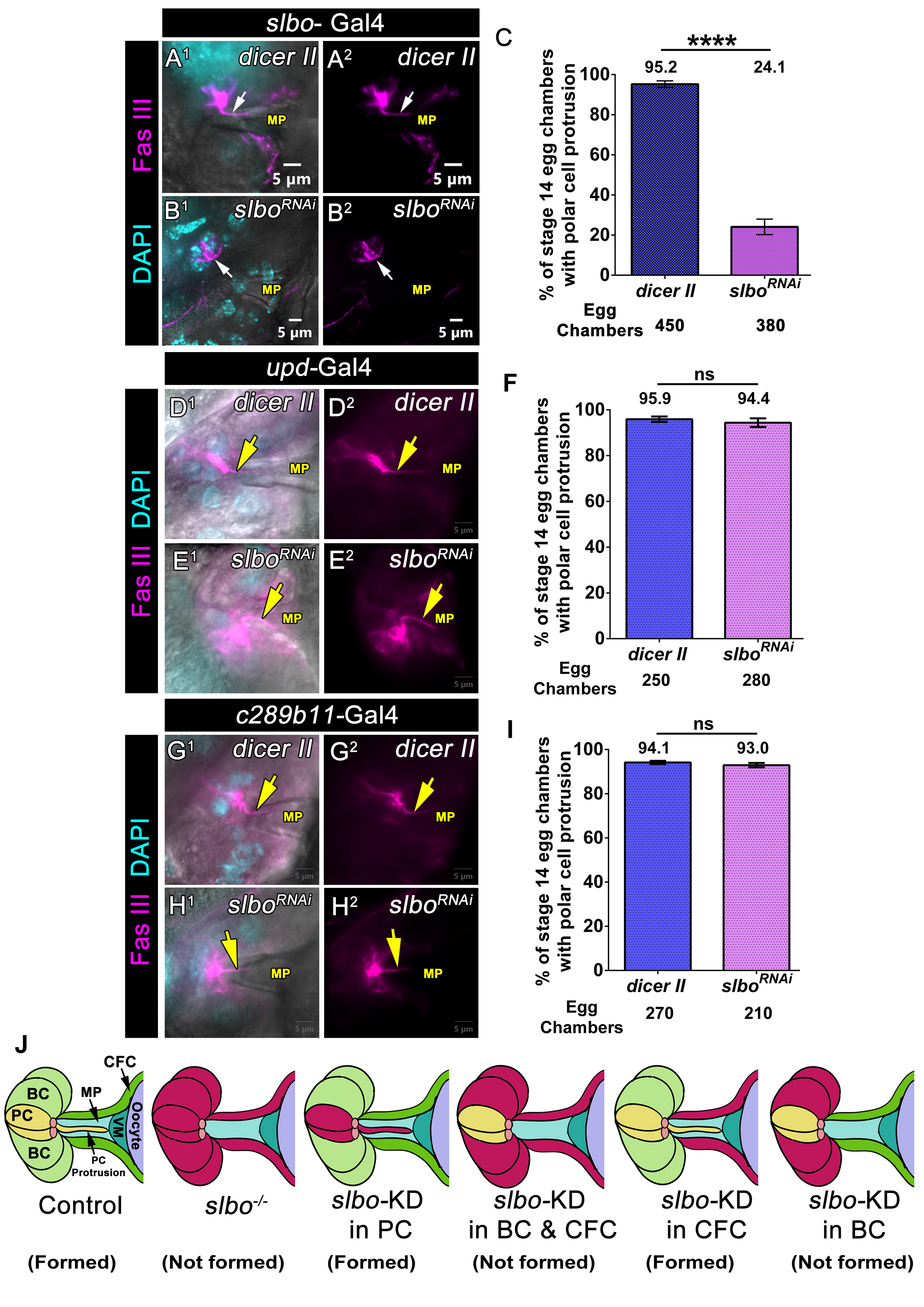

Supplement: S7 Fig — (A1–C) Downregulation of Slbo function in the outer border cells affects polar cell protrusion formation. (A1–B2) Stage 13 egg chamber of indicated genotypes, Fas III (Magenta) and DAPI (Cyan). White arrows indicate polar cell protrusion. (C) Quantification of % of stage 14 egg chambers with polar cell protrusion. Error bars represent SEM, nonparametric t test, ****P < 0.0001. Detailed quantification in S11 Data. (D1–F) Downregulation of Slbo function in the polar cells does not impede polar cell protrusion formation. (D1–E2) Stage 13 egg chamber of indicated genotypes, Fas III (Magenta) and DAPI (Cyan). Yellow arrows indicate polar cell protrusion. (F) Quantification of % of stage 14 egg chambers with polar cell protrusion. Error bars represent SEM, nonparametric t test, ns P > 0.05. Detailed quantification in S11 Data. (G1–I) Downregulation of Slbo function in the centripetal cells does not impede polar cell protrusion formation. (G1–H2) Stage 13 egg chamber of indicated genotypes, Fas III (Magenta) and DAPI (Cyan). Yellow arrows indicate polar cell protrusion. (I) Quantification of % of stage 14 egg chambers with polar cell protrusion. Error bars represent SEM, nonparametric t test, ns P > 0.05. Detailed quantification in S11 Data. (J) Schematic of polar cell protrusion formation at stage 13. Where light green cells are outer border cells, yellow cells are polar cells, dark green cells are centripetal cells, purple color represents oocyte. Please note that downregulation of slbo in the outer border cells impede formation of the polar cell protrusion. (TIFF) [file pbio.3003533.s007.tiff]

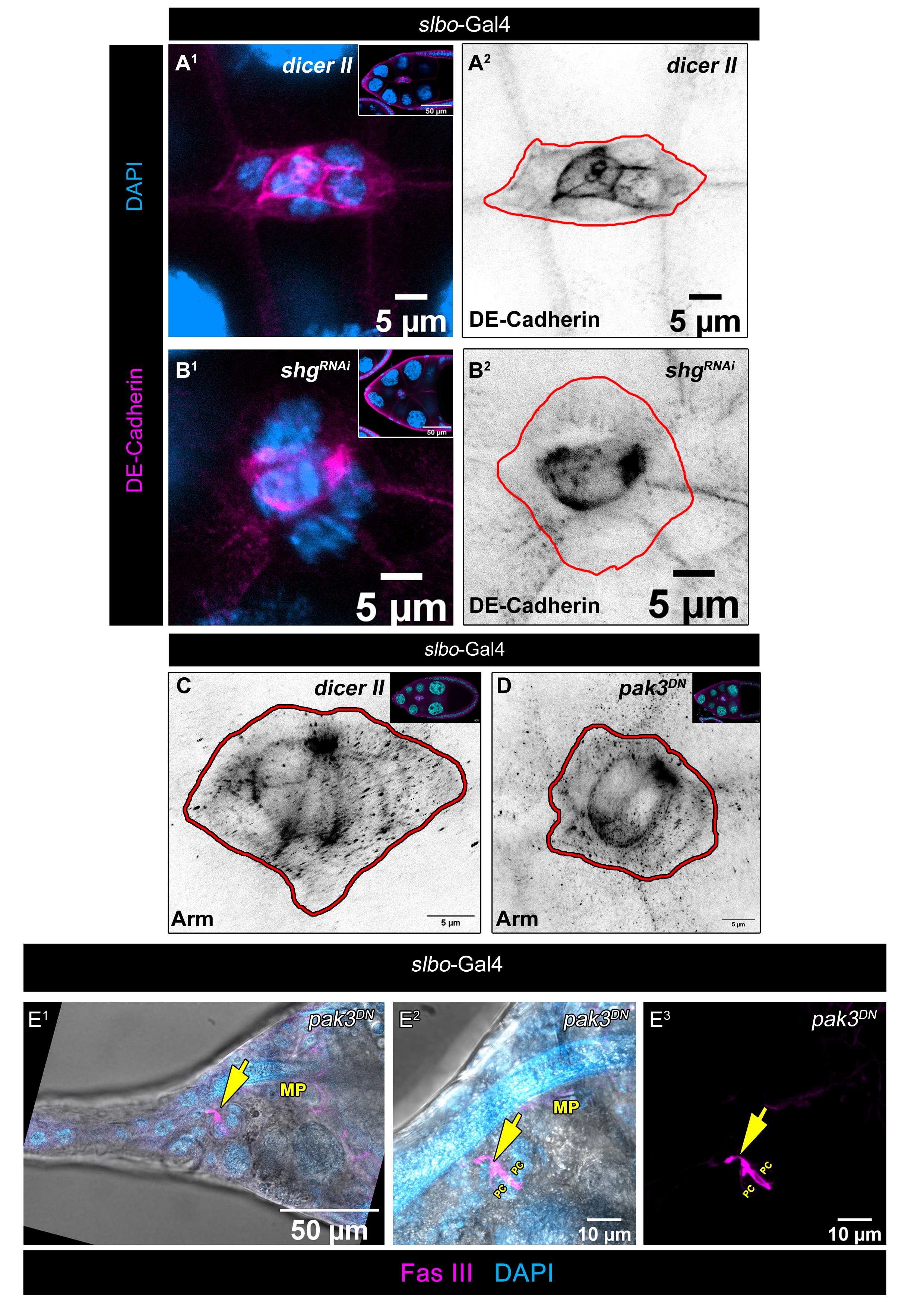

Supplement: S8 Fig — (A1–B1) Downregulation of Shg function in the border cell affects the distribution of DE-Cadherin localization in the migratory cluster. The DE-Cadherin is localized in the PC-PC, PC-BC, BC-BC junction (A1, A2), which is completely reduced in between BC-BC and BC-PC in the Shg-depleted border cell cluster (B1, B2). DE-Cadherin (Magenta in A1, B1 and Black in A2, B2), DAPI (Cyan) inset box represents reference egg chamber of indicated genotypes, red line indicates the outline of BC cluster. (C, D) Downregulation of Pak3 function in the border cell affects the distribution of Armadillo localization in the migratory cluster. The Armadillo is localized in the PC-PC, PC-BC, BC-BC junction (C), which is altered in the Pak3-depleted border cell cluster (D), Armadillo (Black), red line indicates the outline of BC cluster, inset box represents reference egg chamber of indicated genotypes, showing Armadillo (Magenta) and DAPI (Cyan). (E1–E3) Stage 13 egg chamber of indicated genotype, Fas III (Magenta), and DAPI (Cyan), yellow arrows indicate polar cell protrusion. (TIFF) [file pbio.3003533.s008.tiff]

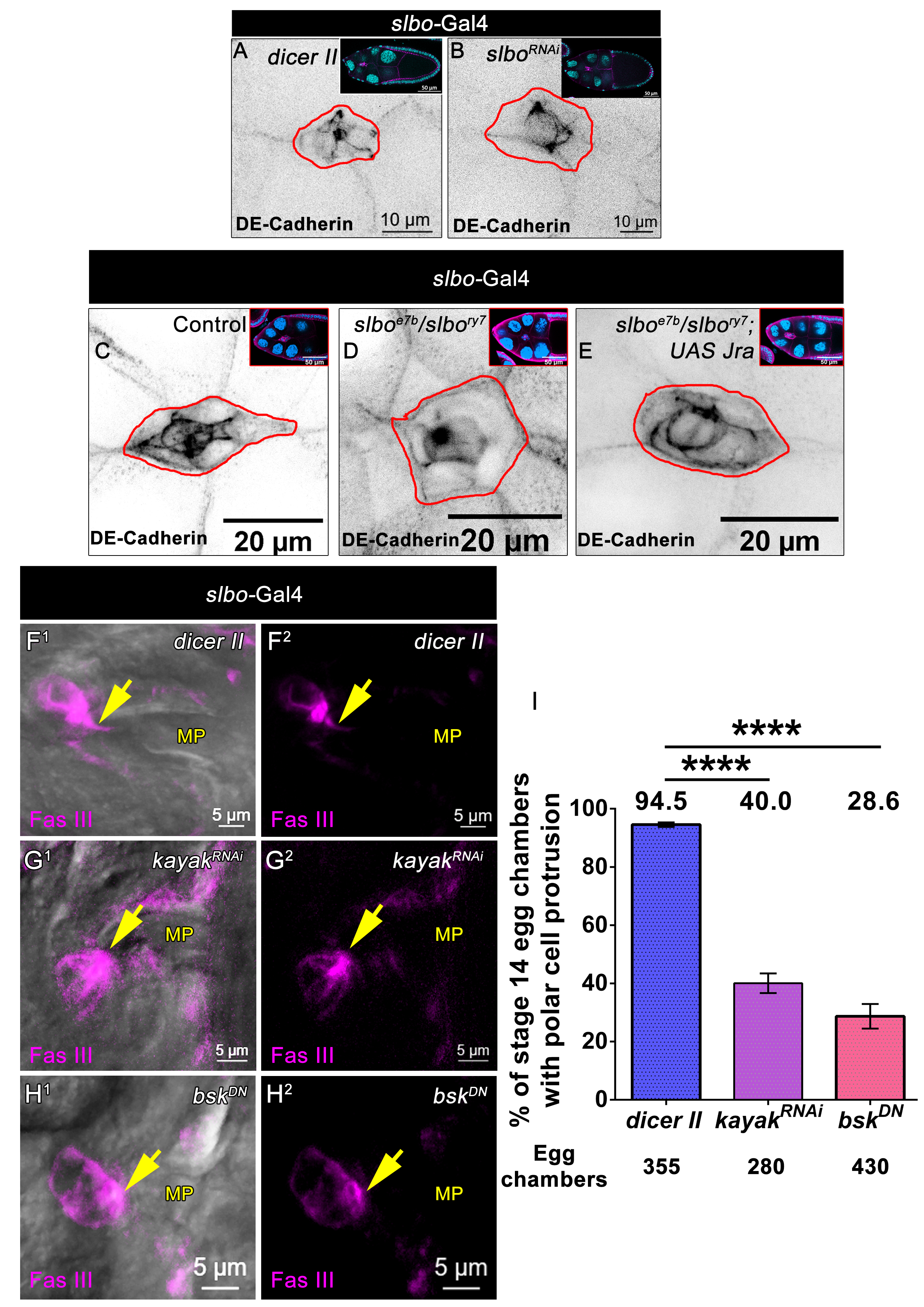

Supplement: S9 Fig — (A, B) Downregulation of Slbo function in the border cell affects the distribution of DE-Cadherin localization in the migratory cluster. The DE-Cadherin is localized in the PC-PC, PC-BC, BC-BC junctions (A), which is altered in Slbo-depleted border cell cluster (B), DE-Cadherin (Black), red line indicates the outline of BC cluster, inset box represents reference egg chamber of indicated genotypes, showing DE-Cadherin (Magenta) and DAPI (Cyan). (C–E) Overexpression of Jra in slbo mutant border cells rescues the polarity of the border cell cluster. The DE-Cadherin localization is altered in the slbo mutant border cell cluster (D), and it rescues in overexpression of Jra in slbo mutant cluster (E). DE-Cadherin (Black), red line indicates the outline of BC cluster, inset box represents reference egg chamber of indicated genotypes, showing DE-Cadherin (Magenta) and DAPI (Cyan). (F1–I) Downregulation of JNK signaling in the border cells affects polar cell protrusion formation. (F1–H2) Stage 13 egg chamber of indicated genotypes, Fas III (Magenta) and DAPI (Cyan), yellow arrows indicate polar cell protrusion. (I) Quantification of % of stage 14 egg chambers with polar cell protrusion. Error bar SEM, nonparametric t test, ****P < 0.0001. Detailed quantification in S12 Data. (TIFF) [file pbio.3003533.s009.tiff]

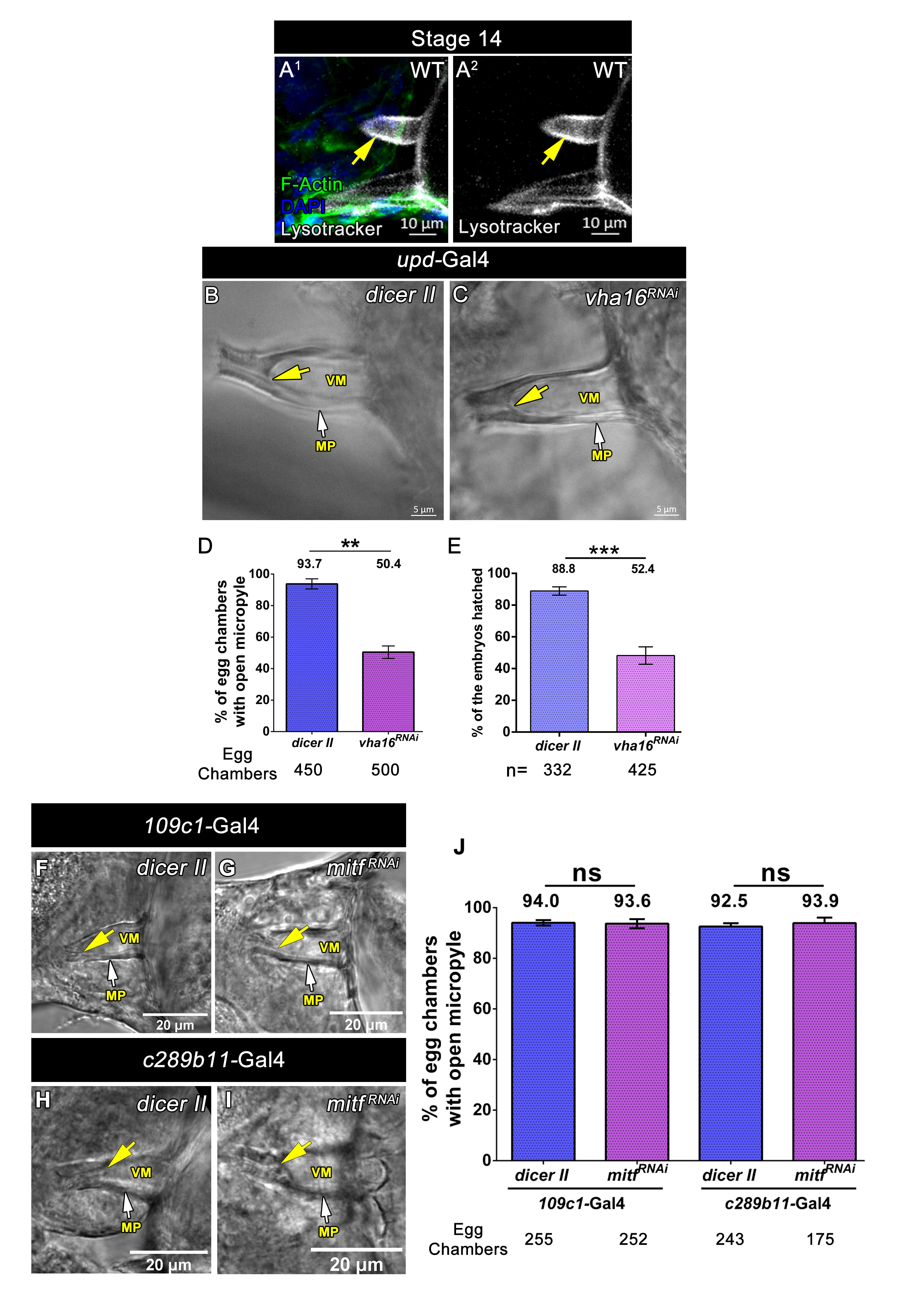

Supplement: S10 Fig — (A1–A2) The micropyle cone is highly acidic in nature. Stage 14 egg chamber was stained by lysotracker, where lysotracker in white, F-Actin in green, and DAPI in blue, the yellow arrows indicate the micropyle cone structure. (B–D) Downregulation of Vha-16 function in the polar cells affects micropyle channel opening. (B, C) DIC images of Stage 14 egg chambers of indicated genotypes, yellow arrows indicate micropyle channel, and white arrows indicate the micropyle cone. (D) Quantification of % of stage 14 egg chamber with open micropyle. Error bars represent SEM, nonparametric t test, **P < 0.01. Detailed quantification in S13 Data. (E) Quantification of % of embryos hatched after 30 hours of egg laying. Error bars represent SEM, nonparametric t test, ***P < 0.001. Detailed quantification in S13 Data. (F–J) Downregulation of Mitf function in the BCs and CFCs does not impede micropyle channel opening. (F–I) DIC images of Stage 14 egg chamber of indicated genotypes, yellow arrows indicate micropyle channel, and white arrows indicate the micropyle cone. (J) Quantification of % of stage 14 egg chambers with open micropyle. Error bars represent SEM, nonparametric t test, ns P > 0.05. Detailed quantification in S13 Data. (TIFF) [file pbio.3003533.s010.tiff]
