# Supplementary material for: Polar cell membrane nanotubes containing microtubules and acidic vesicles render Drosophila eggs fertile
Source: PLoS Biol. 2025 Dec 2;23(12):e3003533. doi: 10.1371/journal.pbio.3003533 (PMC12697985; doi:10.1371/journal.pbio.3003533)
Supplement: S1 Table — (PDF) [file pbio.3003533.s011.pdf]

S1 Table : List of Lysosomal proteins

S.No.	Gene Name	Symbol
1	Mitf	*Mitf*
2	Gamma-interferon-inducible lysosomal thiol reductase 1	*GILT1*
3	Autophagy-related 8a	*Atg-8a*
4	Rab7	*Rab7*
5	Vacuolar H^+ ATPase 16kD	*Vha16*
7	Bicaudal D	*BicD*
8	deep orange	*dor*
9	spoonbill	*spoon*
